# Surfactant–Particle Engineering Hybrids: Emerging Strategies for Enhancing Solubility and Oral Bioavailability of Poorly Water-Soluble Drugs

**DOI:** 10.3390/pharmaceutics18010037

**Published:** 2025-12-26

**Authors:** Kyeong-Soo Kim, Hyuk Jun Cho, Fakhar Ud Din, Jung Hyun Cho, Han-Gon Choi

**Affiliations:** 1 Department of Pharmaceutical Engineering, Gyeongsang National University, 33 Dongjin-ro, Jinju 52725, Republic of Korea; soyoyu79@gnu.ac.kr; 2 College of Pharmacy, Keimyung University, 1095 Dalgubeoldaero, Dalseo-gu, Daegu 42601, Republic of Korea; hjcho89@kmu.ac.kr; 3 Department of Pharmacy, Quaid-I-Azam University, Islamabad 45320, Pakistan; fudin@qau.edu.pk; 4 Department of Pharmaceutical Engineering, Dankook University, 119 Dandae-ro, Dongnam-gu, Cheonan 31116, Republic of Korea; 5 College of Pharmacy, Hanyang University, 55 Hanyangdaehak-ro, Sangnok-gu, Ansan 15588, Republic of Korea

**Keywords:** poorly water-soluble drugs, surfactant–particle hybrid systems, oral drug delivery, supersaturation stabilization, pharmacokinetic enhancement

## Abstract

**Background/Objectives:** The poor aqueous solubility of many therapeutic compounds remains a key barrier to achieving optimal oral bioavailability. While traditional formulation strategies—such as surfactant-based solubilization, nanocrystals, and amorphous solid dispersions—have yielded varying degrees of success, they are often limited by poor physical stability, high excipient loads, inconsistent absorption, and safety concerns associated with long-term surfactant exposure. To address these challenges, this review evaluates surfactant–particle hybrid drug delivery systems as a next-generation platform for enhancing the oral delivery of poorly water-soluble drugs. **Methods:** A comprehensive literature analysis was conducted to examine the mechanistic foundations, formulation techniques, and translational hurdles associated with these hybrid systems. Representative in vitro and in vivo case studies were critically reviewed to assess performance consistency, particularly with respect to dissolution enhancement, supersaturation stabilization, and permeability modulation. Consideration was also given to manufacturing feasibility, excipient safety, scalability, and regulatory constraints. **Results:** Findings indicate that surfactant–particle hybrids provide synergistic benefits by integrating solubilization and stabilization functions with tailored particle design. These systems have shown consistent improvements in pharmacokinetic profiles and drug absorption across diverse drug candidates. However, limitations remain, including challenges in long-term physical stability and excipient compatibility that must be addressed for broader application. **Conclusions:** Surfactant–particle hybrid systems offer a versatile and promising approach to overcoming the limitations of poorly soluble drugs. With careful attention to formulation optimization and regulatory compliance, they have the potential to serve as a transformative platform in future oral drug delivery strategies.

## 1. Introduction

The oral administration of pharmaceuticals remains the most preferred route for drug delivery due to its convenience, cost-effectiveness, and patient compliance. However, a persistent and widely recognized challenge in oral drug development is the poor aqueous solubility of a large proportion of active pharmaceutical ingredients (APIs). Approximately 40–60% of new chemical entities (NCEs) identified through high-throughput screening are classified as poorly water-soluble [1,2]. According to the Biopharmaceutics Classification System (BCS), drugs in Class II (low solubility, high permeability) and Class IV (low solubility, low permeability) suffer from dissolution-limited absorption, resulting in erratic pharmacokinetics, suboptimal bioavailability, and inconsistent therapeutic outcomes [3]. This issue not only complicates drug development and dosage form design, but also influences clinical translation, particularly for molecules with narrow therapeutic windows or food-dependent absorption profiles.

Given the critical link between solubility and systemic exposure, numerous formulation strategies have been developed to address this bottleneck, ranging from chemical modifications (e.g., prodrug synthesis, salt formation) to physical and formulation-based approaches such as particle size reduction, amorphization, lipid-based delivery, and the use of solubilizing excipients like surfactants, polymers, and cyclodextrins [4,5,6,7,8]. Among these, particle engineering and surfactant-based solubilization have independently emerged as two of the most widely applied and regulatory-accepted tools for oral formulation development [9,10]. Particle engineering methods, such as micronization, nanonization, spray-drying, and amorphous solid dispersion (ASD) formation, aim to increase surface area, reduce crystallinity, and improve dissolution rate as governed by the Noyes–Whitney equation [11,12]. Surfactants, on the other hand, function primarily by improving wettability, lowering interfacial tension, forming micelles above the critical micelle concentration (CMC), and interacting with biological membranes to facilitate absorption [13,14].

However, despite the well-documented successes of each approach, both strategies suffer from intrinsic limitations when applied in isolation. Particle engineering techniques often lead to issues related to physical instability, including aggregation, Ostwald ripening, and polymorphic transitions that can diminish in vivo performance and complicate long-term storage [15,16]. Likewise, surfactants, especially when used at high concentrations, may induce gastrointestinal toxicity, promote drug precipitation upon dilution, or interfere with drug release kinetics due to excipient–drug interactions [17,18]. Moreover, in vivo dilution of micelles can result in sub-CMC conditions, causing drug recrystallization and loss of solubilization advantage—a phenomenon that is frequently underestimated during formulation screening.

To overcome these individual shortcomings, there is a growing interest in hybrid formulation strategies that integrate surfactant systems with particle engineering techniques. These surfactant–particle engineering hybrids are designed to combine the structural advantages of engineered particles with the interfacial and solubilizing functionalities of surfactants, thus maximizing dissolution and absorption while minimizing the drawbacks of each component [19,20,21]. For instance, surfactant-coated nanocrystals exhibit improved wettability, controlled particle dispersion, and reduced aggregation compared to uncoated counterparts [22]. Similarly, incorporating surfactants during wet granulation or solid dispersion formation has been shown to improve compressibility, flowability, and redispersibility, contributing to both pharmacokinetic enhancement and manufacturability [23]. This synergistic integration is not merely additive; it offers mechanistic complementarity that can enhance supersaturation maintenance, inhibit precipitation, modulate membrane permeability, and support biopharmaceutic predictability.

An important differentiator of surfactant–particle hybrids from other advanced drug delivery systems—such as liposomes, polymeric micelles, or solid lipid nanoparticles—is their relative simplicity and scalability. These systems can be manufactured using well-established unit operations (e.g., wet milling, spray-drying, high-shear granulation), often without the need for sterile processing or complex synthesis pathways [24]. The availability of GRAS (Generally Recognized As Safe)-status surfactants listed in the FDA (Food and Drug Administration)’s Inactive Ingredient Database further reduces the regulatory burden and accelerates product development [25]. This positions hybrid systems as a practical and economically viable solution for industry-scale applications, including both generic reformulations and new molecular entity (NME) development pipelines.

Recent studies also highlight the ability of these hybrids to enhance in vitro–in vivo correlation (IVIVC), which is critical for predicting human pharmacokinetics and obtaining biowaivers during regulatory submissions [26,27]. For example, hybrid systems using micronized salts with surfactant-assisted granulation have demonstrated bioequivalence to reference listed drugs while maintaining consistent dissolution across physiologically relevant pH ranges [28]. Such outcomes underscore the translational potential of these platforms—not just for solubility enhancement but also for enabling cost-effective development, lifecycle management, and patient-centric formulation design.

Despite this progress, the current literature often discusses particle engineering or surfactant-based methods in isolation, without adequately exploring the interplay between them in a mechanistic or design-oriented context. Recent reviews typically focus on either nanocrystal stabilization, micellar solubilization, or polymer–surfactant interactions, but rarely provide a unified perspective on how these elements can be rationally integrated [29,30]. Furthermore, there remains a lack of systematic frameworks guiding the selection and optimization of hybrid platforms based on API typology, biopharmaceutical challenges, and manufacturing feasibility.

This review seeks to address that gap by offering a comprehensive, design-forward perspective on surfactant–particle engineering hybrids. We aim to (i) critically evaluate the physicochemical roles and mechanistic functions of commonly used surfactants, (ii) summarize state-of-the-art particle engineering technologies, (iii) elucidate the synergistic principles that underlie hybrid system performance, and (iv) analyze recent case studies across various formulation modalities—including granules, nanocrystals, solid dispersions, and self-emulsifying systems. We further discuss the industrial, biopharmaceutical, and regulatory implications of adopting hybrid strategies, while identifying areas where predictive modeling, real-time analytics, and safety assessment must evolve to support broader adoption.

Ultimately, this review positions hybrid strategies not merely as a workaround for solubility challenges, but as a next-generation paradigm in oral drug delivery—one that integrates physicochemical engineering with translational viability. As new therapeutics continue to challenge formulation scientists with low solubility, permeability, or stability, surfactant–particle hybrids may hold the key to unlocking robust, scalable, and patient-aligned dosage forms. By bridging mechanistic understanding with industrial applicability, we aim to provide a roadmap for more rational and effective formulation development in the era of complex APIs.

To differentiate this review from prior literature, we explicitly integrate the mechanistic principles of surfactant function with particle engineering strategies under a unified framework. While earlier reviews tend to focus on individual techniques (e.g., lipid-based carriers or nanosuspensions), this work emphasizes the synergy between surfactant selection, particle morphology, and hybridization approaches. Furthermore, we incorporate regulatory, biopredictive, and personalized formulation perspectives, offering a holistic roadmap for future hybrid drug delivery development.

## 2. Surfactants in Drug Formulation

### 2.1. Classification and Functional Roles

Surfactants are amphiphilic molecules that have become indispensable in modern pharmaceutical formulations for poorly water-soluble drugs. Their molecular structure, which includes both hydrophilic and lipophilic domains, enables them to localize at the solid–liquid interface, where they can significantly reduce surface tension, promote wetting, and facilitate the solubilization of hydrophobic compounds through micelle formation—thereby enhancing both dissolution and oral absorption [31,32].

Surfactants are broadly categorized based on the ionic nature of their polar head group: non-ionic, anionic, cationic, and zwitterionic. Among these, non-ionic surfactants are the most frequently employed in oral dosage forms due to their relatively low toxicity, chemical and thermal stability, and compatibility with a broad range of APIs and excipients [33]. Representative examples include Polysorbate 80 (Tween 80), Cremophor EL, Solutol HS15, and PEGylated surfactants, all of which are either GRAS listed or included in the FDA Inactive Ingredient Database [34,35]. These surfactants are effective solubilizers primarily because they form micelles above their critical micelle concentration (CMC), encapsulating hydrophobic drug molecules in their lipophilic core to enhance apparent solubility and maintain supersaturation during gastrointestinal transit [36].

However, the role of surfactants extends well beyond micellization. One key mechanism is adsorption onto solid drug particles, which enhances wetting and reduces the energy barrier for particle–liquid contact [37]. This is especially beneficial for APIs with high hydrophobicity and large contact angles. For example, Polysorbate 80 has been shown to significantly shorten the lag time during dissolution of poorly wettable drugs such as curcumin and celecoxib, enabling faster initial dissolution and improved bioavailability [38].

In particle engineering systems such as nanocrystals, surfactants play an equally critical role by stabilizing nanoscale drug particles during and after processing. Their adsorption on particle surfaces prevents aggregation via steric hindrance or electrostatic repulsion, thereby preserving high surface area and colloidal stability [39]. In such formulations, surfactants are often co-formulated with hydrophilic polymers like PVP or HPMC to create dual stabilization systems, improving both shelf-life and redispersibility in aqueous media [40].

A practical summary of commonly used surfactants—covering HLB (Hydrophilic-Lipophilic Balance) values, physicochemical behavior, and regulatory classification—is provided in Table 1 to assist formulators in rational surfactant selection based on drug properties and target delivery profile.

Despite their widespread utility, the use of ionic surfactants (e.g., SLS for anionic and CTAB for cationic) is often limited in oral formulations. While these agents demonstrate potent surface activity and permeability enhancement, they are also associated with higher cytotoxicity and mucosal irritation, especially under chronic exposure conditions [41,42]. Nonetheless, they are selectively employed in applications requiring efflux transporter inhibition—particularly of P-glycoprotein (P-gp)—and in enhancing paracellular permeability for challenging molecules like peptides or BCS Class IV drugs [43,44]. Their inclusion must therefore be carefully justified and supported by toxicological evaluation and regulatory acceptability.

A growing strategy to mitigate toxicity while maximizing performance is the use of surfactant blends. For example, binary combinations such as Polysorbate 80 and Labrasol have demonstrated synergistic effects in reducing the CMC and improving micelle stability [45]. These blends allow for reduced total surfactant load while preserving or enhancing solubilization performance, an important consideration in formulations targeting chronic administration. However, these strategies also introduce complexity, as they require careful preformulation assessment to avoid excipient–excipient incompatibilities, unintended crystallization, or undesirable release kinetics [46].

Importantly, within the context of surfactant–particle engineering hybrids, surfactants take on a more integrated and multifunctional role. They are no longer limited to solubilizing agents but function as interfacial modifiers, precipitation inhibitors, and even mucoadhesive agents, depending on their chemical structure and incorporation method. For instance, in nanocrystal systems or amorphous solid dispersions, surfactants can modulate the surface energy of drug particles, influence redispersibility, and delay recrystallization under gastrointestinal conditions [39,40].

Yet, the utility of surfactants must be balanced against their dose-dependent risks. At high concentrations, certain surfactants have been shown to disrupt epithelial tight junctions or increase non-specific permeability, potentially allowing unwanted absorption of toxins or allergens [42]. Others, particularly polyoxyethylene-based surfactants such as Cremophor EL, are vulnerable to oxidative degradation, forming peroxides that may compromise drug stability and patient safety [34,35]. These risks highlight the need for careful concentration control, robust excipient screening, and context-specific justification—especially for long-term oral therapies or formulations targeting sensitive populations.

In conclusion, surfactants are highly versatile and strategically valuable excipients in the formulation of poorly water-soluble drugs. Their classification, physicochemical properties, and mechanistic roles must be understood not only in isolation but also in conjunction with drug characteristics and formulation goals. In emerging hybrid delivery systems, surfactants are increasingly recognized as enabling components that bridge the gap between particle engineering and in vivo performance. Their judicious use—guided by mechanistic insight and regulatory considerations—can play a pivotal role in developing scalable, effective, and biopharmaceutically robust drug products.

### 2.2. Mechanistic Benefits and Limitations

The efficacy of surfactants in enhancing drug bioavailability arises from a combination of interfacial, colloidal, and biological mechanisms. Four major pathways are widely recognized: (i) enhancement of surface wettability, (ii) micellar solubilization of hydrophobic compounds, (iii) modulation of intestinal membrane permeability, and (iv) inhibition of efflux transporters such as P-glycoprotein (P-gp) [47,48].

Among these, micelle formation remains the most extensively investigated mechanism. Once the concentration of surfactant surpasses the critical micelle concentration (CMC), molecules self-assemble into micelles, with hydrophobic drug molecules encapsulated in their core. This micellar encapsulation dramatically increases apparent solubility, enabling supersaturation of lipophilic APIs and maintaining them in a dissolved state throughout transit in the gastrointestinal tract [48,49]. However, the efficiency and reproducibility of this process are highly dependent on the drug’s affinity for the micellar core, micelle stability under physiological dilution, and interaction with bile salts and intestinal contents.

Certain surfactants—particularly non-ionic and amphiphilic block copolymers—also exert biological effects by modulating membrane fluidity and inhibiting drug efflux mechanisms. For example, Cremophor EL and Polysorbate 80 have been shown to inhibit P-gp-mediated transport, enhancing systemic absorption of substrates such as paclitaxel and loperamide [47,50,51]. However, the clinical translation of these effects remains inconsistent. In vitro inhibition of P-gp does not always correlate with improved in vivo pharmacokinetics, due to confounding factors such as enzyme-mediated clearance, mucosal variability, and transporter redundancy in the intestinal wall. These inconsistencies highlight the need for mechanistic validation beyond cell-based assays, especially when surfactants are relied upon to bypass biological barriers.

Despite these benefits, surfactants possess several critical limitations that can compromise formulation robustness, clinical safety, and regulatory success.

First, at high concentrations, surfactants may disrupt epithelial tight junctions, leading to non-specific increases in membrane permeability [42,52]. This can result in gastrointestinal (GI) irritation, local inflammation, or even unintended absorption of toxins or allergens. Such effects are especially concerning for vulnerable populations including pediatrics, geriatrics, or patients with inflammatory bowel disease. Chronic exposure to surfactants in long-term oral therapies demands precise excipient control and risk-based safety evaluations.

Second, polyoxyethylene-based surfactants (e.g., Cremophor EL, PEG 400) are prone to oxidative degradation, particularly under conditions of elevated humidity, temperature, or in the presence of reactive APIs. This degradation generates peroxides and aldehydes, which not only compromise drug stability but may also interact with APIs to form reactive impurities, raising both toxicological and regulatory red flags [53,54]. For liquid and semi-solid formulations, such oxidative stress is a leading cause of shelf-life reduction and formulation failure.

A third limitation is micelle dilution. In vivo, upon oral administration, surfactants are rapidly diluted by gastrointestinal fluids. If the concentration falls below the CMC, micelles disassemble and drug molecules previously solubilized may precipitate or recrystallize [55]. This leads to a sharp decline in free drug concentration, undermining the bioavailability advantage gained during formulation. Well-documented cases with itraconazole and ritonavir show that supersaturation often collapses within 30–60 min post-administration, resulting in marked reductions in plasma exposure [56]. Moreover, in early-phase clinical studies, ritonavir-containing SEDDS (Self-Emulsifying Drug Delivery System) formulations exhibited up to 40% intersubject variability in supersaturation duration and bioavailability, complicating efforts to demonstrate bioequivalence in regulatory bridging studies [57].

These findings underscore the fragility of micelle-based strategies in dynamic physiological conditions. Overreliance on micelle-mediated solubilization, without accounting for in vivo dilution kinetics, excipient–drug interaction dynamics, and interindividual variability, can lead to formulation failures that are not predicted by standard in vitro testing.

To mitigate these limitations, recent advances focus on integrating surfactants within particle-engineered platforms. For example, surfactant-coated nanocrystals using low-HLB surfactants can retain dispersion and solubilization characteristics with significantly reduced surfactant load [58]. Because the surfactant remains associated with the particle surface rather than being free in solution, the risk of micelle disassembly upon dilution is minimized. This approach also offers benefits in terms of physical stability, lower toxicity, and regulatory acceptability—especially when GRAS-status surfactants are selected.

**Figure 1:**
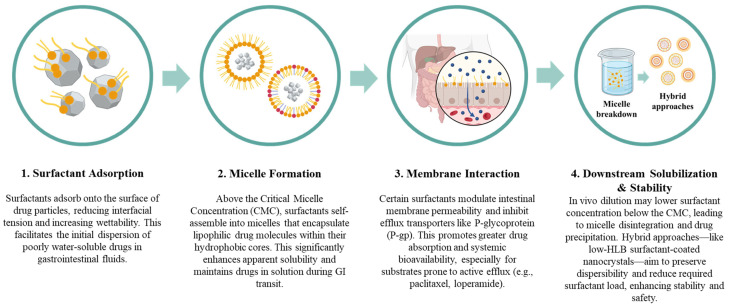
Mechanistic role and limitations of surfactants in drug delivery: A four-stage overview. This schematic outlines the key mechanistic pathways through which surfactants enhance oral drug bioavailability—beginning with surface adsorption and micelle formation, followed by membrane interaction, and concluding with downstream challenges such as micelle instability and precipitation. It also highlights emerging hybrid formulation strategies designed to overcome these limitations and improve formulation robustness.

A visual synthesis of these mechanisms—including surface adsorption, micelle formation, membrane interaction, and downstream risks such as micelle instability and drug precipitation—is presented in Figure 1. This figure also introduces how hybrid formulation strategies are being developed to mitigate these limitations and enhance overall formulation robustness.

Such strategies reflect a broader shift toward hybrid systems, wherein surfactants are not simply solubilizing agents but mechanistically integrated components of the delivery matrix. For instance, in amorphous solid dispersions, surfactants contribute to both interfacial stabilization and nucleation inhibition, delaying recrystallization under intestinal conditions. In SEDDS–particle hybrids, surfactants embedded in solid carriers can enable reconstitution without loss of colloidal stability. These innovations demonstrate that understanding not just what surfactants do, but how and where they act within a formulation, is essential for building predictive, patient-adapted, and regulation-compliant oral dosage forms [19,58,59].

In sum, while the mechanistic versatility of surfactants remains a cornerstone of modern formulation science, their application in isolation carries inherent risks and limitations. Hybrid strategies that embed surfactants into structurally engineered systems offer a compelling path forward—one that balances biopharmaceutical performance with manufacturability, safety, and translational reliability.

## 3. Particle Engineering Techniques

### 3.1. Core Technologies

Particle engineering plays a pivotal role in modern formulation design by tailoring the physicochemical and biopharmaceutical properties of drug particles to enhance their solubility, dissolution rate, and oral bioavailability. The primary goal is to increase surface area, modulate solid-state characteristics, and optimize interfacial behavior—thereby overcoming the intrinsic limitations of hydrophobic, poorly water-soluble active pharmaceutical ingredients (APIs) [60].

#### 3.1.1. Micronization

Micronization, primarily via jet milling or air-jet comminution, remains the most widely applied industrial technique for size reduction. It effectively reduces particle sizes into the 1–10 μm range, significantly increasing surface area and enabling faster initial wetting [61]. However, the technique has notable drawbacks: irregular particle morphology, electrostatic charging, and poor powder flow can hinder downstream manufacturability [62,63]. These issues have led to tablet capping, blending failures, and content uniformity deviations in commercial settings—often necessitating post-processing via granulation or dry coating.

Furthermore, micronization does not inherently alter the interfacial energy of hydrophobic APIs. As a result, many micronized drugs still exhibit poor wettability in vivo, despite showing acceptable dissolution profiles in vitro. This divergence highlights the disconnect between surface area and interfacial activity, and underscores the need for surface-active excipients to achieve sustained biopharmaceutical performance.

#### 3.1.2. Nanocrystals

To overcome these shortcomings, nanocrystal technology has gained prominence. Nanocrystals—produced via top-down methods (e.g., wet media milling) or bottom-up approaches (e.g., antisolvent precipitation)—offer several advantages: (i) rapid dissolution due to increased surface area (per the Noyes–Whitney equation); (ii) controlled and often spherical particle morphology; (iii) surface modifiability via polymers or surfactants [64,65].

Yet, nanocrystals are thermodynamically unstable. Over time, they are susceptible to Ostwald ripening, aggregation, and sedimentation, especially under stress conditions like temperature fluctuation or mechanical vibration [22,66]. This instability not only threatens long-term shelf life but also affects in vivo performance. Indeed, nanoformulations with >90% dissolution in vitro have, in some cases, demonstrated less than 20% C_max_ improvement due to aggregation during storage or precipitation upon dilution in the GI tract. Moreover, mechanical processing steps such as wet milling or spray granulation can induce polymorphic transitions that alter dissolution kinetics and result in batch variability—a critical factor in regulatory rejections and failed scale-ups [67].

Thus, despite their promise, nanocrystals are not universally applicable. Their success depends on solid-state characterization, stabilization strategy, and a predictive understanding of API–excipient interactions throughout the product lifecycle.

#### 3.1.3. Spray-Drying and Freeze-Drying

Solution-based techniques like spray-drying and freeze-drying provide an alternative route for producing tailored particles, particularly in the form of amorphous solid dispersions (ASDs) or polymeric encapsulates.

Spray-drying is widely used due to its scalability and control over particle morphology, but thermal stress can degrade heat-sensitive APIs [68]. For example, degradation exceeding 5% potency loss has been reported in industrial-scale production of poorly stable APIs, necessitating process redesign or inclusion of thermal protectants. Solvent compatibility and residual solvent levels are also critical regulatory considerations.

Freeze-drying offers superior protection for thermolabile drugs and allows formation of highly porous structures favorable for reconstitution. However, it is energy-intensive, time-consuming, and prone to structural collapse during storage if residual moisture exceeds optimal thresholds [68,69]. Consequently, its use is typically limited to high-value, low-dose therapeutics where product stability justifies the cost.

#### 3.1.4. Amorphous Solid Dispersions (ASDs)

ASDs are a cornerstone in modern particle engineering for bioavailability enhancement. They involve molecular dispersion of the API within a polymeric carrier above the glass transition temperature, resulting in a high-energy amorphous form with improved apparent solubility [70]. However, such systems are thermodynamically metastable and prone to recrystallization or phase separation, especially under humid storage or in the presence of ionic excipients [71].

Real-world regulatory submissions have documented shelf-life reductions when ASDs are stored above 30 °C/65% RH, indicating a disconnect between lab-based stability data and distribution conditions. Recent innovations include the incorporation of surfactants (e.g., TPGS (D-α-Tocopheryl Polyethylene Glycol 1000 Succinate), Cremophor RH40) to improve interfacial stability and suppress nucleation [72]. These combinations can delay crystallization and improve physical robustness. However, surfactant–polymer compatibility is essential, as some surfactants may plasticize the matrix and accelerate phase separation if improperly matched.

A comparative overview of these techniques—highlighting particle size range, thermodynamic stability, scalability, cost-efficiency, and common drug targets—is presented in Table 2, offering a foundation for formulation-by-design strategies in particle engineering.

#### 3.1.5. Limitations and Rationale for Integration

While each particle engineering technology provides unique advantages, none is universally sufficient. Micronized and nanonized APIs may still exhibit poor wettability or recrystallize rapidly during dissolution, undermining in vivo performance. Similarly, ASDs with excellent in vitro profiles may fail stability tests or exhibit variable bioavailability under fed vs. fasted conditions.

These recurring limitations form a compelling rationale for integration with surfactant systems. Surfactants can address both interfacial energy barriers and physical instability, enabling synergistic control over solubility, redispersibility, precipitation inhibition, and even mucoadhesion. In this context, surfactants are no longer viewed merely as solubilizers, but rather as functional enablers embedded within the particle matrix. This hybridization offers a pathway to more predictable, scalable, and regulatory-compliant oral formulations, particularly for drugs at the edge of BCS Class II/IV boundaries.

### 3.2. Effect of Particle Size on Dissolution and Absorption

Particle size reduction is a fundamental strategy in formulation science, primarily aimed at improving drug dissolution and absorption. According to the Noyes–Whitney equation, the dissolution rate is directly proportional to the surface area of drug particles in contact with the medium [11,73]. Reducing particle size increases this surface area, thereby accelerating dissolution for poorly water-soluble drugs. However, in practical systems, this theoretical advantage is modulated by factors such as particle agglomeration, wettability, polymorphic stability, and excipient interactions [74].

#### 3.2.1. Micronized Particles: Surface Area vs. Functionality

Micronization reduces drug particles to the range of 1–10 μm and remains widely adopted due to its scalability and cost-effectiveness. While surface area increases substantially, micronized particles often exhibit electrostatic charges, poor flowability, and aggregation tendencies, which can compromise blend uniformity and tablet compaction [75]. Additionally, micronized particles—especially those with high hydrophobicity—can resist wetting due to their high surface energy. This paradox highlights a common challenge: increased surface area does not guarantee increased wettability or bioavailability.

To counteract this, surface modification is often required. The use of surfactants or hydrophilic polymers can improve wettability, prevent agglomeration, and stabilize the drug–excipient interface. For instance, incorporation of Polysorbate 80 or hydrophilic binders during dry granulation has been shown to improve uniformity and dissolution consistency in micronized formulations [61,76].

#### 3.2.2. Nanocrystals: Supersaturation and Metastability

Nanocrystal formulations further reduce particle size to below 1000 nm, often reaching the 100–300 nm range. At this scale, the diffusion layer thickness decreases, leading to higher dissolution rates and the possibility of achieving supersaturation in the gastrointestinal tract [77]. This enables drug concentrations to transiently exceed their equilibrium solubility, improving absorption.

However, this supersaturated state is thermodynamically metastable and highly sensitive to environmental conditions. Upon dilution with gastric or intestinal fluids, the concentration of stabilizers (e.g., surfactants, polymers) may fall below the threshold needed to inhibit nucleation and crystal growth, leading to rapid precipitation [78]. The collapse of supersaturation not only reduces bioavailability but also introduces inter-subject variability, making clinical outcomes less predictable.

Recent studies have highlighted the importance of hybrid strategies, where nanocrystal production is coupled with surface-active agents. For example, wet milling in the presence of Polysorbate 80 or Soluplus has resulted in nanocrystals with enhanced colloidal stability, improved GI retention, and increased permeability [79]. These approaches not only preserve particle size during storage but also enhance mucosal interaction and absorption in vivo.

#### 3.2.3. Amorphous–Crystalline Hybrids and Spray-Dried Systems

Another method of leveraging particle size involves the formation of amorphous–crystalline hybrid systems, often via spray-drying. In such systems, APIs are co-dissolved with surfactants and polymers, producing partially crystalline matrices that combine dissolution efficiency with physical stability [80]. These hybrids act as controlled dissolution reservoirs and have been shown to outperform fully crystalline or fully amorphous forms under fed and fasted conditions alike.

#### 3.2.4. Shifting the Limiting Step: From Dissolution to Permeability

One of the most compelling benefits of particle size reduction—particularly at the nanoscale—is its ability to shift the rate-limiting step of absorption. In many BCS Class II drugs, dissolution is the primary barrier. By significantly accelerating dissolution, nanoformulations can transform these APIs into permeability-limited systems. This shift allows membrane interaction and translocation to become the dominant determinant of bioavailability, particularly when supported by surfactants that modulate membrane fluidity and tight junction permeability [81].

This effect is particularly useful in enhancing absorption across challenging biological barriers like the intestinal epithelium, nasal mucosa, or pulmonary surfaces. However, this benefit is also context-dependent, influenced by patient-specific factors (e.g., GI motility, bile salt concentration), formulation pH, and co-administered excipients.

#### 3.2.5. Manufacturing and Regulatory Considerations

Despite their biopharmaceutical advantages, finely reduced particles introduce significant manufacturing and regulatory challenges. Smaller particles exhibit greater surface energy and reactivity, increasing risks of content non-uniformity, electrostatic charging, and loss during filtration [82]. During tableting, over-compression of nanosized materials may cause capping or lamination. Moreover, the high-energy surfaces of nanocrystals are more prone to uncontrolled interactions with moisture, excipients, or packaging materials, especially when ionic surfactants or hygroscopic carriers are used [83].

These factors complicate scale-up and long-term quality assurance, prompting regulatory agencies to demand extensive solid-state characterization, dissolution robustness testing, and accelerated stability studies for approval. In several documented cases, nanocrystal-based products failed to meet bioequivalence or stability thresholds due to recrystallization or unexpected drug–excipient interactions.

#### 3.2.6. Strategic Implications and Conceptual Integration

**Figure 2:**
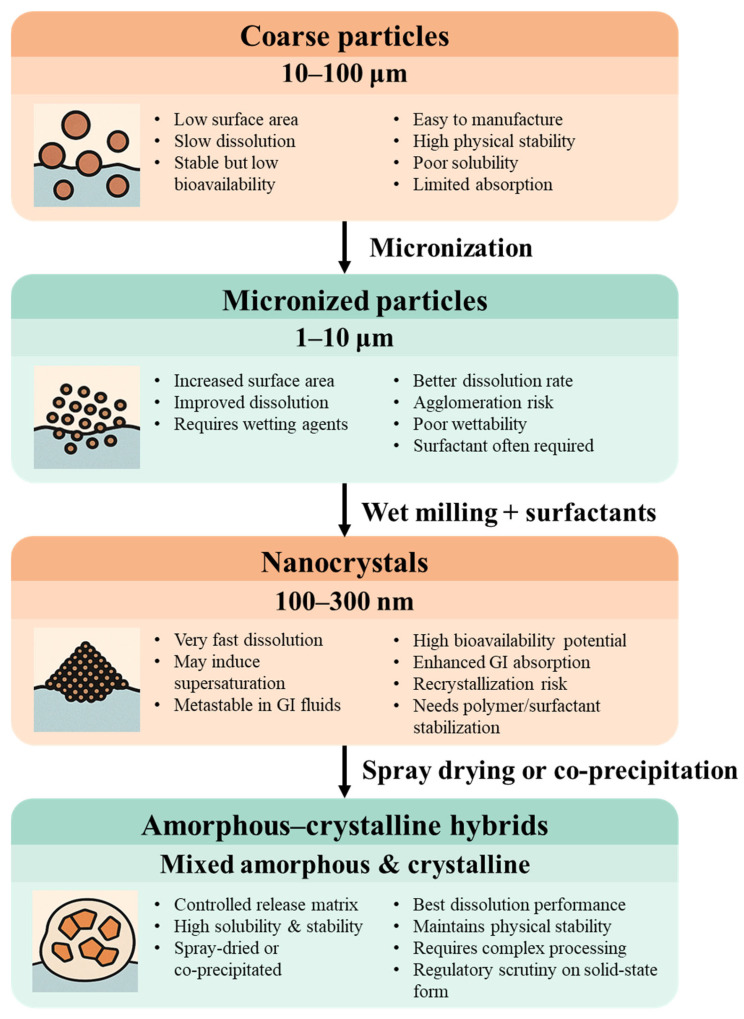
Conceptual progression of particle size reduction and its impact on drug dissolution and formulation properties. This schematic illustrates the biopharmaceutical evolution of drug particles from coarse forms to micronized particles, nanocrystals, and ultimately amorphous–crystalline hybrids. As particle size decreases, dissolution rate and absorption potential improve; however, each stage introduces new challenges in physical stability, wettability, and manufacturing complexity. Stabilization strategies such as surfactant incorporation and polymeric support are noted as critical at advanced stages to maintain supersaturation and formulation robustness.

Figure 2 conceptually illustrates how particle size reduction—from coarse powders to micronized forms, nanocrystals, and amorphous–crystalline hybrids—influences dissolution behavior, absorption kinetics, and formulation complexity. As particle size decreases, the dissolution rate improves, but so does the demand for stabilization strategies, manufacturing control, and in vivo performance modeling.

Ultimately, while particle engineering significantly improves formulation flexibility and performance, it is not a panacea. Its limitations in physical stability, manufacturability, and long-term predictability make it clear that size reduction must be integrated into multifunctional formulation platforms. This sets the stage for Section 4, which introduces surfactant–particle hybrids as next-generation delivery systems that offer superior control over dissolution, permeability, and regulatory viability [84,85,86,87,88,89].

## 4. Hybrid Strategies: Surfactant–Particle Engineering Integration

### 4.1. Why Combine?

While both surfactants and particle engineering techniques independently improve drug solubility through distinct mechanisms, they also exhibit mechanistic and operational limitations when applied in isolation—particularly for high-risk, low-solubility compounds.

Surfactants function primarily through micellization, surface tension reduction, and interfacial adsorption, enabling rapid solubilization and improved wetting of hydrophobic drugs [90]. However, these benefits are often transient and concentration-dependent. In the gastrointestinal tract, surfactants are diluted below their critical micelle concentration (CMC), leading to micelle disintegration and drug precipitation. Moreover, high surfactant loads—especially of polyoxyethylene-based or ionic types—can induce membrane irritation, tight junction disruption, and toxicity, raising concerns for chronic oral administration.

Conversely, particle engineering techniques such as nanocrystal formation, micronization, and amorphous solid dispersion (ASD) aim to improve dissolution through physical transformation of the drug [91,92]. By reducing particle size or altering crystallinity, these methods increase surface area and apparent solubility. However, they do not inherently enhance wetting or membrane permeation, and often suffer from instability issues such as aggregation, recrystallization, or phase separation. These physical changes may compromise shelf-life, bioavailability, and scale-up reproducibility.

Hybrid strategies address these challenges by integrating surfactants into particle-engineered systems, thereby leveraging the complementary advantages of both approaches. Particle engineering offers structural control—modifying particle size, shape, and solid-state properties—while surfactants act at the interface, regulating solubility, permeability, and supersaturation maintenance [10,20,93]. This synergy enables multimodal enhancement across the entire oral delivery process: (i) Enhanced wettability and redispersibility in aqueous environments; (ii) micelle-assisted solubilization and stabilization of hydrophobic drugs; (iii) inhibition of precipitation or crystallization upon GI dilution; (iv) improved membrane interaction, permeability, and lymphatic transport; (v) reduction in required surfactant load due to stabilization via engineered particles.

Such benefits are particularly impactful for BCS Class II and IV drugs, which are limited by both low solubility and low permeability. In these cases, surfactant–particle hybrids allow for targeted improvement of both rate-limiting steps in absorption. Additionally, because surfactants are embedded within the particle matrix or localized at the surface, their systemic exposure is minimized—mitigating toxicity concerns and aligning with regulatory safety thresholds.

From a development perspective, hybrid strategies offer enhanced design flexibility and formulation robustness. By adjusting the ratio and type of surfactant–polymer–particle interactions, developers can fine-tune critical quality attributes (CQAs) such as dissolution rate, supersaturation duration, physical stability, and inter-batch uniformity. This adaptability supports Quality by Design (QbD) frameworks, facilitating risk-based design space exploration and scale-up predictability.

Moreover, the total excipient burden—a key determinant of regulatory and clinical acceptability—can often be reduced in hybrid systems. Because the combined mechanism is more efficient, less surfactant is needed to achieve a given biopharmaceutical effect, improving patient safety and manufacturing efficiency.

**Figure 3:**
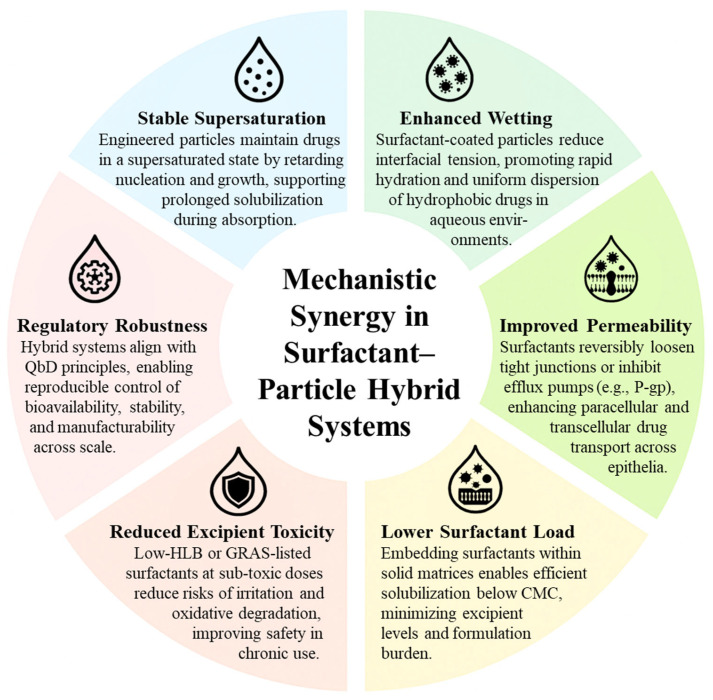
Conceptual framework of synergistic benefits derived from surfactant–particle hybrid systems. The central hybrid core enables a cascade of effects, enhancing both pharmacokinetic predictability and formulation robustness while minimizing toxicity risks.

These integrative effects are synthesized in Figure 3, which conceptually maps the interaction of surfactant and particle engineering functions across the oral drug delivery pathway—from initial dispersion and solubilization to intestinal permeation and systemic absorption. The figure illustrates how hybridization provides both depth (mechanistic coverage) and breadth (multifactorial enhancement)—resulting in more predictable pharmacokinetics, reduced formulation failure rates, and increased clinical success for poorly soluble compounds.

In summary, hybrid strategies represent not just a combination of excipients and processes, but a strategic convergence of material science, pharmacokinetics, and regulatory compliance. They mark a critical evolution in oral drug delivery, enabling the development of formulations that are scientifically rational, clinically effective, and industrially viable.

### 4.2. Representative Hybrid Techniques

Hybrid platforms differ in their structural complexity and manufacturing feasibility, but they converge on a central principle: combining physical design of the drug particle with interfacial modulation at the biological interface.

#### 4.2.1. Micronization

Surfactant-assisted wet granulation incorporates surface-active agents into the granulation fluid to improve the wettability and dispersion of hydrophobic APIs during oral solid dosage manufacturing. This method has demonstrated success with poorly water-soluble drugs such as telmisartan, ritonavir, and naproxen, using surfactants like Polysorbate 80, PEG 400, or sodium lauryl sulfate (SLS) to enhance dissolution rates [94,95,96,97]. The mechanism involves the uniform deposition of surfactant on drug particle surfaces within the granule matrix, thereby facilitating rapid wetting during disintegration and minimizing hydrophobic agglomeration during dissolution.

A key advantage of this approach lies in its compatibility with conventional high-shear granulation equipment, which allows for seamless integration into existing manufacturing lines without significant capital investment. Additionally, surfactants can function as binder plasticizers, improving granule compressibility and tablet robustness, which contributes to better downstream processability. Importantly, this method often requires only low surfactant concentrations, reducing the risk of exceeding regulatory excipient thresholds and improving its suitability for chronic or high-dose formulations.

Despite these benefits, the technique is sensitive to process parameters. The success of surfactant-assisted granulation depends heavily on factors such as liquid distribution uniformity, granulation endpoint control, and drying kinetics. Inadequate mixing may lead to heterogeneous surfactant coverage, creating intra-batch dissolution variability. Conversely, excessive surfactant loading or prolonged granulation can result in over-wetting, leading to granule densification, disintegration delay, or even processing failures. Additionally, some surfactants—particularly PEG 400—may migrate to granule surfaces during drying, increasing stickiness, moisture uptake, and compromising stability under humid storage conditions.

From a translational perspective, multiple studies have confirmed that surfactant-assisted wet granules outperform both conventionally granulated and dry-blended formulations in dissolution performance [94,95,96,97]. However, there are documented cases where this advantage diminished after accelerated stability testing, likely due to API–surfactant phase separation or moisture-induced structural changes. These outcomes highlight the importance of incorporating stability screening and solid-state characterization early in the formulation process.

In conclusion, surfactant-assisted wet granulation represents a practical and scalable hybridization strategy that enhances the interfacial behavior of hydrophobic APIs without overhauling manufacturing infrastructure. Nevertheless, its success requires careful optimization of surfactant type, concentration, and process parameters to ensure robust, reproducible performance across development stages and commercial production.

#### 4.2.2. Surfactant-Coated Nanocrystals

Nanocrystals, typically generated through wet media milling, antisolvent precipitation, or high-pressure homogenization, offer substantial improvements in dissolution for poorly soluble drugs. However, without stabilization, they are prone to aggregation, Ostwald ripening, and crystal growth during storage or upon reconstitution. To address this, surfactants are employed to coat nanocrystal surfaces, providing both physical stability and biopharmaceutical enhancement via interfacial modulation.

Surfactant coatings confer multiple functional benefits: they improve zeta potential, reduce interparticle attraction, and enhance redispersibility—especially critical after drying or compaction. Furthermore, certain surfactants enhance mucoadhesion and membrane interaction, increasing the potential for localized delivery or improved cellular uptake. For example, PLGA (Poly(lactic-co-glycolic acid)) nanocrystals coated with surfactants demonstrated superior lung deposition and cellular internalization in pulmonary delivery models [98]. Similarly, curcumin and silymarin nanocrystals stabilized with Poloxamer 407 or sodium deoxycholate showed markedly improved cellular uptake, likely due to surfactant-facilitated membrane permeation [99].

More advanced approaches employ multilayer coating strategies, where surfactants are combined with polymers such as PVP or TPGS to create a composite interface that offers pH-triggered release, enzyme resistance, and targeted delivery in segment-specific regions of the GI tract. Such designs can also protect labile APIs from degradation and are particularly valuable for oral delivery of peptides or herbal actives. Surfactant-coated nanocrystals are increasingly being adapted into orodispersible tablets and fast-dissolving films, enabling easier administration for pediatric, geriatric, or dysphagic patients [100,101].

However, these benefits come with formulation and manufacturing challenges. The performance of surfactant-coated nanocrystals is highly dependent on coating uniformity, surfactant desorption kinetics, and long-term stability. Incomplete or non-uniform coating may allow crystal bridging or aggregation during storage. Furthermore, in physiological environments, surfactant desorption—particularly in the presence of bile salts or dilution media—can lead to loss of stabilization and in vivo precipitation, thereby diminishing bioavailability.

Toxicological concerns also arise from excessive surfactant loading, especially for pulmonary or parenteral applications where localized concentrations can exceed safe thresholds. In such settings, off-target uptake or epithelial irritation has been reported, necessitating careful excipient screening during formulation development.

From a manufacturing standpoint, ensuring reproducible coating at commercial scale requires precise control over parameters such as mixing shear, surfactant-to-drug ratio, and post-drying conditions. Batch-to-batch variability in zeta potential and redispersibility has been directly linked to inconsistency in these parameters. Moreover, even when initial stabilization is achieved, accelerated storage studies have revealed signs of partial recrystallization or Ostwald ripening, underlining the need for robust shelf-life modeling and real-time stability monitoring.

In conclusion, surfactant-coated nanocrystals represent a versatile hybrid platform that combines the advantages of particle size reduction with interfacial engineering to overcome multiple formulation barriers. However, successful translation into clinical and commercial use depends on balanced design—achieving stability and efficacy without compromising safety or manufacturability—and on implementing rigorous quality control systems to ensure coating integrity throughout the product lifecycle.

#### 4.2.3. Solid Dispersions with Surfactants

Solid dispersions—especially those prepared by hot-melt extrusion (HME) or spray-drying—represent a widely adopted strategy to enhance solubility and dissolution of poorly water-soluble drugs. The integration of surfactants into these systems provides a further layer of functional control, enabling improvements in drug–polymer miscibility, supersaturation maintenance, and in vivo predictability. Surfactants such as Cremophor RH40, Solutol HS15, and Vitamin E TPGS have been extensively employed in ternary solid dispersions, where they serve roles beyond mere solubilization [102,103].

Mechanistically, surfactants can reduce interfacial tension between the drug and polymer, facilitating more uniform dispersion at the molecular or nanodomain level. They also modulate drug mobility and physical aging behavior by interacting with the polymer matrix. In some systems, surfactants increase the glass transition temperature (Tg) by forming specific intermolecular associations that restrict segmental motion, thereby improving amorphous phase stability. Critically, surfactant-assisted solid dispersions have demonstrated the ability to maintain supersaturation for extended durations in biorelevant media such as FaSSIF or FeSSIF, leading to reduced food effect and more consistent pharmacokinetics [104].

However, these benefits are highly formulation-dependent, and improper surfactant selection may introduce new risks. Certain surfactants act as plasticizers, reducing Tg and accelerating recrystallization or phase separation under stress conditions such as high humidity or temperature. Additionally, surfactant–polymer incompatibility can result in microscale demixing during storage, which undermines the system’s stability and alters its dissolution profile. In some spray-dried formulations, surfactant migration to the surface has been observed during storage, leading to moisture uptake, stickiness, or changes in redispersion behavior—even within a few months under accelerated conditions.

From a manufacturing standpoint, the thermomechanical behavior of surfactants can significantly influence process reproducibility. In HME, certain surfactants reduce melt viscosity excessively, altering extrusion torque and residence time. Surfactants with low thermal stability may degrade during processing, forming reactive byproducts that compromise the safety and shelf-life of the final product. In spray-drying, volatile or low-boiling-point surfactants may partially evaporate, resulting in batch-to-batch variability unless drying parameters are precisely optimized. Moreover, surfactant inclusion often increases the viscosity of the feed solution, complicating atomization and residual solvent removal.

From a translational perspective, while surfactant-assisted solid dispersions remain among the most promising formulations for oral delivery of hydrophobic drugs, their success hinges on more than just initial dissolution enhancement. Real-world viability depends on a careful balance of polymer–surfactant compatibility, storage stability, and manufacturing control, ensuring that in vitro benefits translate into robust clinical performance.

#### 4.2.4. Self-Emulsifying Drug Delivery Systems (SEDDS) with Particle Engineering

Self-emulsifying drug delivery systems (SEDDS) are isotropic mixtures of oils, surfactants, and cosolvents that spontaneously form oil-in-water emulsions upon contact with gastrointestinal fluids. They offer a well-established platform for enhancing the oral absorption of lipophilic drugs, particularly those with poor aqueous solubility. However, liquid SEDDS are associated with challenges such as physical instability, phase separation, low portability, and limited patient acceptability, especially in chronic regimens or pediatric settings.

To overcome these limitations, recent advancements have focused on integrating particle engineering to convert liquid SEDDS into solid-state formulations, including spray-dried microparticles, adsorbed powders, or granular intermediates [105,106]. These hybridized forms preserve the solubilization benefits of SEDDS while significantly improving physical robustness, dose uniformity, and formulation flexibility.

Solidification techniques typically involve spray-drying the liquid SEDDS onto porous inorganic carriers (e.g., Neusilin, Aerosil), or co-precipitating the drug–lipid mixture into a dry powder using antisolvent or adsorption-based processes. For example, ritonavir-loaded solid SEDDS incorporating Poloxamer 188 exhibited improved redispersibility, droplet size uniformity, and dissolution rate compared to the conventional liquid form [107]. Furthermore, these hybrid systems have demonstrated the ability to enhance lipid-mediated lymphatic uptake, a pathway that allows partial bypass of hepatic first-pass metabolism, offering distinct pharmacokinetic advantages for cyclosporine, tacrolimus, and similar drugs [108].

Nevertheless, several formulation and performance challenges persist. The ability of solid SEDDS to fully and rapidly re-emulsify after storage or tableting is not guaranteed. Compression stress during tableting may deform or collapse the porous structure of the carrier, reducing emulsion reconstitution efficiency. Additionally, migration of surfactant or lipid to the particle surface during storage can lead to droplet size heterogeneity, reduced bioavailability, and altered release kinetics. Moisture uptake further complicates stability, potentially inducing premature phase separation or partial crystallization of the API within the surfactant–lipid matrix.

From a manufacturing perspective, spray-drying and adsorption processes must be optimized to ensure uniform drug distribution, adequate powder flow, and low agglomeration risk. The choice of carrier material, surfactant properties, and drying conditions significantly impact key quality attributes such as reconstitution time, droplet size post-redispersion, and compression compatibility. Batch-to-batch variability in emulsion performance has been reported when process conditions are not tightly controlled [109].

Despite these limitations, particle-engineered SEDDS offer a strategically flexible platform for formulating lipophilic APIs into solid oral dosage forms such as tablets and capsules. This enables improved patient compliance, industrial scalability, and regulatory acceptability. However, long-term performance depends on rigorous stability testing under realistic storage conditions and quantitative assessment of reconstitution kinetics and bioavailability retention from lab scale through to commercial deployment.

#### 4.2.5. Nanostructured Lipid–Surfactant Hybrid Systems

Nanostructured lipid carriers (NLCs) represent an advanced class of lipid-based nanocarriers that incorporate a blend of solid and liquid lipids, providing enhanced drug loading, physical stability, and release control compared to first-generation solid lipid nanoparticles. Recent innovations have expanded their potential through surface functionalization with surfactants, such as Polysorbate 80, TPGS, and Cremophor EL, forming a lipid core–surfactant shell structure that enhances both bioavailability and mucosal penetration [110].

This hybrid design enables dual-mode solubilization—lipophilic drugs can partition into the lipid matrix, while amphiphilic or interface-seeking molecules interact at the surfactant-coated surface. For example, quercetin-loaded NLCs stabilized with Tween 80 demonstrated improved nanoparticle dispersion stability and enhanced epithelial permeability, likely due to surfactant-mediated modulation of tight junctions [111]. Similarly, resveratrol-loaded NLCs coated with Poloxamer 407 showed increased in vivo anti-inflammatory activity, underscoring the therapeutic synergy between surfactant-driven permeability enhancement and lipid-based encapsulation [112].

The design flexibility of these systems extends to targeted delivery, wherein the surfactant layer composition can be tailored to achieve site-specific uptake. For instance, incorporation of bile salt surfactants supports hepatotropic delivery, while cationic surfactants enhance pulmonary or intracellular targeting. These strategies expand NLC applicability to challenging indications and routes beyond conventional oral and dermal delivery.

However, successful implementation requires careful management of several formulation-specific limitations. One critical concern is surfactant desorption in systemic circulation, which can result in premature nanoparticle aggregation, opsonization, and loss of targeting efficiency. Moreover, some synthetic surfactants used for functionalization exhibit dose-dependent cytotoxicity or immunogenicity, especially in chronic administration scenarios, raising significant regulatory scrutiny [113].

Storage stability is another major constraint. The interplay between surfactant type, lipid polymorphism, and storage conditions may lead to phase separation, lipid crystallization, or alteration in drug release kinetics. Such changes can undermine therapeutic consistency, particularly when formulations are exposed to temperature fluctuations or high humidity during distribution.

From a manufacturing perspective, scaling up surfactant-coated NLCs while maintaining tight control over particle size, surface charge, and drug encapsulation efficiency poses a technical challenge. Even minor deviations in mixing intensity, cooling rates, or surfactant concentration can cause batch-to-batch variability. This necessitates the deployment of process analytical technologies (PAT) and stringent quality control protocols to ensure product reproducibility and regulatory compliance.

In summary, surfactant-functionalized NLCs represent a highly versatile hybrid platform that effectively combines lipid-based solubilization with surfactant-driven targeting and stabilization mechanisms. Their potential to enable precise, site-specific drug delivery—alongside enhanced permeability and controlled release—makes them a compelling choice for next-generation formulations. Nonetheless, clinical translation and industrial scalability require deliberate balancing of functionality, safety, and process robustness to ensure alignment with regulatory standards and therapeutic goals.

#### 4.2.6. Spray Freeze-Drying with Surfactant Co-Precipitation

Spray freeze-drying (SFD) is a specialized particle engineering technique that integrates feed solution atomization, rapid cryogenic freezing, and low-pressure sublimation to produce highly porous, low-density particles. This process uniquely generates spherical microstructures with large surface area, offering rapid dissolution and dispersion characteristics ideal for poorly soluble or thermally sensitive drugs. When surfactants are co-precipitated in the feed solution, the resulting API–surfactant hybrids benefit from enhanced reconstitution, wetting, and interfacial stabilization [114].

One of the key advantages of SFD lies in its thermal gentleness. Unlike spray-drying or hot-melt extrusion, SFD avoids high temperatures, making it well-suited for thermolabile molecules such as peptides, proteins, and vaccines. For instance, voriconazole–SLS–mannitol hybrid particles prepared via SFD for pulmonary delivery exhibited significantly improved aerosolization efficiency and dissolution rate compared to conventionally milled powders [115]. Similar benefits have been observed in nasal vaccine formulations utilizing lecithin-based surfactants, where the porous structure enabled fast mucosal dispersion and reconstitution within seconds [116].

Mechanistically, surfactant co-precipitation in SFD contributes by forming a distributed interfacial layer within the porous particle matrix. This layer facilitates rapid penetration of aqueous media upon administration, minimizing the lag phase and preventing drug aggregation. The core–shell-like distribution—with drug-rich regions enveloped by surfactant domains—enables both supersaturation maintenance and surface energy reduction, promoting improved in vivo solubilization.

However, this technique is not without critical limitations. The need for cryogenic media (e.g., liquid nitrogen) and vacuum sublimation contributes to high operational costs, low throughput, and process complexity, which collectively hinder scalability. Additionally, droplet size variability during atomization can lead to broad particle size distribution and inconsistent porosity, directly affecting aerodynamic performance and dissolution uniformity. Uneven surfactant distribution within the matrix can also create concentration gradients, leading to instability or dose inconsistency across the batch.

From a stability standpoint, the high surface area that benefits dissolution also increases susceptibility to moisture uptake and oxidative degradation, particularly when using polyunsaturated lipids or oxidation-prone surfactants. In such cases, incorporation of antioxidants or moisture-barrier packaging may be necessary to preserve shelf-life.

Despite these challenges, SFD holds considerable promise for non-aggregating, surfactant-functionalized dosage forms, particularly in pulmonary, nasal, sublingual, or buccal delivery routes where rapid onset and dispersion are essential [117]. Its ability to produce high-performance microstructures makes it an attractive platform for targeted, high-value therapeutic applications such as anti-infectives, biologics, and rescue medications.

**Figure 4:**
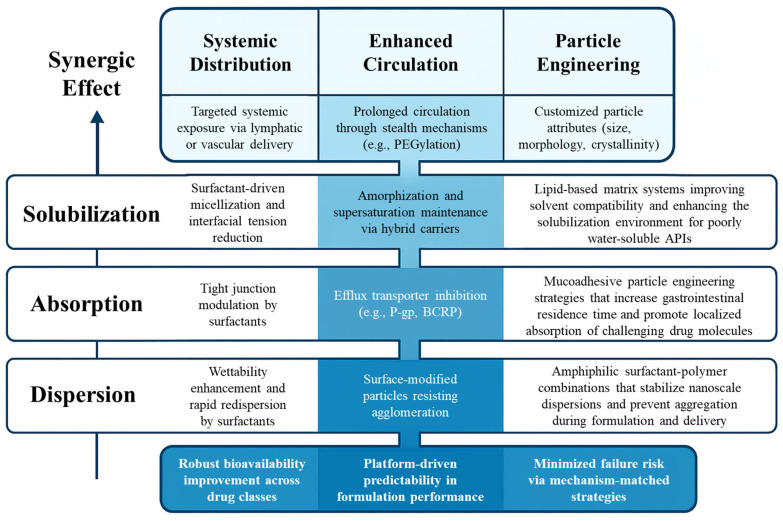
Mechanistic layered model illustrating the synergistic roles of surfactants and particle engineering in hybrid formulation systems. This diagram deconstructs the multilayered interactions between surfactant functionality and particle design across key biopharmaceutical stages, including dispersion, absorption, and solubilization. Each layer represents a mechanistic tier, with upward integration reflecting increasing formulation synergy and systemic impact. The columns highlight distinct contributions—such as enhanced circulation, systemic distribution, and physical particle tuning—demonstrating how hybrid approaches can be strategically leveraged to improve drug delivery efficiency, predictability, and robustness.

A comparative summary of this and other hybrid systems discussed in Section 4.2—including formulation methodology, typical APIs, surfactant classes, and functional outcomes—is presented in Table 3. This tabulation enables strategic platform selection based on drug properties and clinical objectives. Additionally, Figure 4 visualizes a layered mechanistic framework, illustrating how surfactant functionality and particle engineering contributions converge across the delivery cascade—from initial dispersion through membrane permeation to systemic exposure. This schematic clarifies the cumulative effect of hybridization strategies on bioavailability enhancement and formulation robustness.

#### 4.2.7. Comparative Evaluation of Hybrid Systems

To facilitate rational platform selection, Table 4 provides a comparative summary of the hybrid systems described above. Criteria include API load suitability, manufacturing feasibility, stability challenges, and safety considerations. While nanocrystal and SEDDS hybrids offer superior solubility enhancement for high-dose compounds, they require careful surfactant optimization to mitigate toxicity and stability risks. In contrast, micronization and solid dispersion hybrids are more process-friendly but may be limited by moderate drug loading capacity or surfactant compatibility. These comparisons highlight that no single platform is universally optimal; selection must be tailored to drug properties, therapeutic goals, and regulatory constraints.

### 4.3. Strategy Framework for Hybrid Systems

The promise of surfactant–particle hybrid systems lies not merely in their capacity to enhance solubility or permeability, but in their potential to offer tailored, mechanism-driven solutions for diverse drug delivery challenges. However, the absence of a systematic framework to guide platform selection and formulation design has led to empirical, trial-and-error development in many cases. This section presents a critical, design-oriented perspective on how hybrid systems should be chosen and structured, differentiating this review from previous literature that often remains descriptive rather than prescriptive [118].

#### 4.3.1. Aligning Formulation Strategy with API Typology

Designing effective hybrid formulations requires more than identifying whether a drug is poorly soluble or poorly permeable—it demands a granular understanding of the API’s complete biopharmaceutical typology. This includes not only solubility and permeability classifications but also solid-state characteristics (e.g., polymorphic form, crystallinity), wettability, chemical stability, ionization behavior, and lipophilicity. A data-driven API profile should therefore serve as the starting point for surfactant–particle system design, enabling targeted formulation strategies that reduce development cycles and increase translational predictability.

For APIs with high crystallinity and poor wetting properties, such as telmisartan or carvedilol, surfactant-assisted wet granulation can significantly improve dissolution without requiring amorphization or nanonization. In these cases, the surfactant acts primarily as a wettability enhancer and disintegration modulator, facilitating matrix breakdown and uniform dispersion upon ingestion [119]. Such strategies are particularly well-suited to medium-dose oral formulations, where process simplicity and compressibility are critical.

Conversely, APIs that are chemically or physically unstable in crystalline form, such as itraconazole or ritonavir, benefit more from amorphous solid dispersions (ASDs) with integrated surfactants. Here, the combination of polymeric inhibitors (e.g., HPMC, PVP) and interfacial modifiers (e.g., TPGS, Solutol HS15) offers synergistic stabilization against recrystallization while maintaining supersaturation in the gastrointestinal tract [120]. These systems are best applied to APIs with low melting points, high lipophilicity, and known crystallization propensity, which would otherwise fail to maintain solubility post-dosing.

For BCS Class IV drugs, which are simultaneously poorly soluble and poorly permeable, lipid–surfactant hybrid systems such as solid SEDDS or nanostructured lipid carriers (NLCs) offer unique advantages. These platforms leverage the dual mechanism of lipid solubilization and surfactant-mediated permeation enhancement via mucosal interaction or P-gp inhibition [121]. However, they require careful ternary screening of drug–lipid–surfactant compatibility, as misaligned component polarity or phase behavior can result in phase separation, crystalline precipitation, or reduced emulsification efficiency [122].

Ultimately, this alignment of formulation platform with API typology creates a more rational, mechanism-integrated design process. Instead of choosing platforms based solely on solubility classification, developers can now match composite API traits to multi-functional hybrid strategies, improving not only bioavailability but also manufacturability, stability, and regulatory acceptability. This shift represents a critical evolution from descriptive formulation development to a prescriptive, data-informed framework for hybrid system design.

#### 4.3.2. Practical Formulation Constraints

While the theoretical rationale for selecting a surfactant–particle hybrid system may be scientifically sound, real-world formulation success hinges on practical constraints related to manufacturability, scalability, regulatory compliance, and patient-centered considerations. These factors are not peripheral—they must be integrated upfront into the formulation strategy to ensure that laboratory success translates into commercial viability.

For example, spray freeze-drying (SFD) offers unmatched ability to create highly porous, rapidly dispersible particles with excellent dissolution profiles. Yet, it imposes substantial cost, equipment complexity, and cryogenic material handling challenges that restrict its use to low-volume, high-value applications, such as rare disease therapies or biologic-based dosage forms [123]. Conversely, wet granulation and melt granulation approaches, particularly when modified with surfactant pre-blends or in situ precipitation, offer high process scalability, compatibility with existing high-shear granulators, and easier regulatory pathways—making them more attractive for chronic or high-dose oral therapies.

In parallel, the choice between liquid and solid hybrid formats demands careful alignment with API properties, dose strength, and patient demographic. Liquid SEDDS formulations may suffice for lipophilic, low-dose drugs, especially in softgel capsules for adult use. However, when dealing with hygroscopic actives, unstable surfactants, or pediatric populations, transitioning to a solid dosage form—such as a spray-dried SEDDS powder or surfactant-adsorbed granules—may improve stability, palatability, and dose uniformity [124]. Furthermore, liquid forms may pose challenges related to leakage, sedimentation, or excipient–container interactions, particularly in tropical storage conditions.

Formulators must also account for API-specific processing sensitivities, such as thermal or shear degradation, which may preclude the use of hot-melt extrusion or high-shear mixing. In such cases, low-energy particle engineering techniques (e.g., bottom-up nanocrystallization with surfactant capping) can serve as gentler alternatives. Importantly, the regulatory acceptability of certain surfactants (e.g., PEG derivatives, Cremophor types) varies across jurisdictions and must be reconciled with excipient limits, especially for pediatric and chronic indications.

Ultimately, the ideal hybrid system is not only biopharmaceutically effective but also scalable, stable, and regulatorily feasible. A formulation scientist must therefore treat equipment availability, surfactant excipient profiles, and downstream process fit as primary design constraints, not afterthoughts. Only through such a multi-criteria design framework can surfactant–particle hybrid systems move seamlessly from concept to clinic.

#### 4.3.3. Designing with Mechanistic Synergy in Mind

A frequent pitfall in the design of surfactant–particle hybrid systems is the assumption of additive or synergistic performance merely from component combination. However, such formulations often suffer from mechanistic redundancy, interference, or outright antagonism, especially when surfactants and particle systems operate via overlapping or conflicting pathways. Without a clear understanding of these mechanistic interplays, even rationally selected components may yield unexpected instability, diminished performance, or clinical irreproducibility.

For instance, when a high-energy amorphous solid dispersion (ASD) is stabilized by a polymer matrix designed to maintain supersaturation, the inclusion of low-HLB surfactants may inadvertently plasticize the matrix, reduce glass transition temperature (Tg), and accelerate drug recrystallization [125]. Similarly, surfactants like TPGS or Cremophor EL, known for their permeability-enhancing effects, may compete with hydrophilic polymers for hydrogen bonding, weakening matrix cohesion and shortening supersaturation duration. In controlled-release platforms, surfactant migration or diffusion may disrupt diffusion barriers, leading to dose dumping or erratic pharmacokinetics [32].

Therefore, the design of hybrid systems must prioritize mechanistic complementarity rather than component stacking. The following guiding questions should be addressed at the formulation design stage: (i) Does the surfactant enhance wettability or permeability without undermining the physical integrity of the matrix or carrier? (ii) Is the mode of drug stabilization (e.g., micellization, amorphization, crystallization inhibition) reinforced or compromised by the added surfactant? (iii) Do the surfactant and particle engineering elements target distinct rate-limiting steps (e.g., dissolution vs. permeation), or are they redundant? (iv) Are potential antagonisms—such as surfactant-induced phase separation or pH incompatibility—accounted for during screening?

Mechanistic mapping tools—such as QbD-based risk matrices, supersaturation–precipitation assays, and molecular dynamics simulations—can be employed early in development to anticipate and mitigate these issues [126]. These approaches allow for preclinical identification of non-obvious incompatibilities, such as pKa mismatch between surfactant head groups and pH-sensitive carriers, or excipient-induced alteration of drug ionization states.

Furthermore, considering dose timing, site of absorption, and drug metabolism routes can refine mechanistic targeting. For instance, combining a nanocrystal system for rapid dissolution with a mucoadhesive surfactant may be beneficial for drugs with narrow absorption windows, but counterproductive for molecules with extensive first-pass metabolism.

In essence, successful hybrid design should follow a principle not of additive layering, but of functional orchestration. Each component—surfactant, particle form, polymer, lipid—must serve a distinct yet interlocking role within the formulation architecture. Emphasizing this mechanistic synergy minimizes development inefficiencies and increases the likelihood of clinical translation, regulatory approval, and commercial viability.

To further clarify the mechanistic interplay and ensure rational formulation design, we have included Table 5, which categorizes the predominant drug release mechanisms observed across different surfactant–particle hybrid platforms. This summary supports the design considerations discussed above by highlighting how various hybrid types achieve controlled or enhanced release through distinct mechanisms.

#### 4.3.4. Toward a Decision Framework for Hybrid Selection

The selection of an optimal surfactant–particle hybrid system should be guided not by empirical trial-and-error, but by a structured, mechanism-informed decision process. Such a framework must account for critical formulation variables—including API physicochemical properties (e.g., solubility, crystallinity, ionization), biopharmaceutical behavior (e.g., permeability, efflux susceptibility), therapeutic target, and manufacturing feasibility.

**Figure 5:**
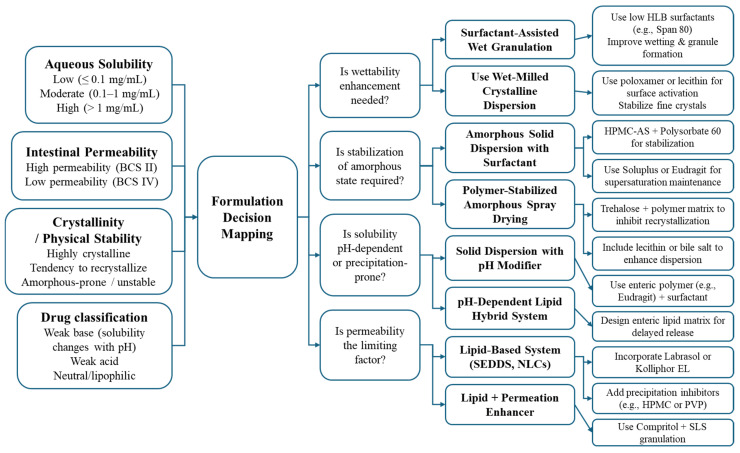
Structured decision tree for rational selection of surfactant–particle hybrid systems. This schematic guides formulation scientists through a stepwise decision-making process based on drug-specific biopharmaceutical properties. The flowchart links key attributes such as solubility class, permeability, crystallinity, and ionization type with suitable hybrid system strategies. Diverging pathways also highlight cases where dual excipient approaches or additional stabilizing mechanisms are warranted to ensure formulation robustness and biopharmaceutical performance.

To support this rational approach, Figure 5 presents a decision tree that maps hybrid formulation strategies against key drug properties. This flowchart helps identify suitable platforms based on solubility–permeability classification, crystallinity, and ionization behavior, aligning each API profile with a platform that offers mechanistic fit, process compatibility, and regulatory feasibility. By visually integrating mechanistic goals with formulation constraints, this tool enables faster and more predictive platform selection.

Such design-oriented strategy facilitates early prioritization of viable formulation paths and supports risk-based design-of-experiments (DoEs) by narrowing down excipient types, processing conditions, and critical quality attributes. More importantly, it minimizes downstream failure caused by mechanistic mismatch or overengineering of platforms that lack biopharmaceutical justification.

In contrast to previous reviews that primarily catalog case studies or platform typologies, this work advances a prescriptive framework for decision-making. It emphasizes design-first formulation thinking, linking theoretical principles to practical translational application in both early-stage screening and late-stage product development [127,128]. This approach provides formulators with a structured methodology, moving beyond descriptive summaries to offer real-world guidance for hybrid system selection and optimization.

## 5. Biopharmaceutical and Industrial Perspectives

### 5.1. In Vitro and In Vivo Evidence

Surfactant–particle hybrid systems have consistently demonstrated improved in vitro performance, particularly in terms of dissolution rate acceleration, supersaturation maintenance, and membrane permeability enhancement, when evaluated in biorelevant media such as FaSSIF and FeSSIF. Surfactants like Polysorbate 80, TPGS, and Cremophor EL contribute to faster wetting and micellization, while engineered drug particles—such as nanocrystals or amorphous solid dispersions—enable rapid disintegration and uniform dispersion [72,129,130].

These biopharmaceutical improvements are not merely theoretical. Several well-documented IVIVC (In Vitro–In Vivo Correlation) studies have revealed strong translation of in vitro enhancements into predictable pharmacokinetic outcomes. For example, a fenofibrate–surfactant nanocrystal system demonstrated a linear correlation between dissolution rate and plasma AUC, underscoring the clinical relevance of dissolution enhancement [131]. Likewise, spray-dried solid dispersions of itraconazole incorporating TPGS sustained supersaturation for over four hours in FaSSIF, with corresponding increases in C_max_ and oral bioavailability observed in both canine and human studies [132].

Beyond dissolution, permeability enhancement has gained increasing attention. Newer tools such as transwell permeability assays and parallel artificial membrane permeability assays (PAMPA) are being employed to capture the mechanistic contributions of surfactants to membrane interaction, tight junction modulation, and P-gp inhibition, which complement particle-based solubilization [133].

However, the translational reliability of these in vitro models depends on thoughtful protocol design. Variables such as surfactant desorption, gastrointestinal dilution effects, and interindividual variability in transporter expression can reduce predictability. For instance, certain supersaturating systems exhibit strong in vitro performance but suffer from precipitation or absorption variability in vivo, particularly in fed-state conditions.

Encouragingly, hybrid systems often exhibit reduced fed/fasted variability, a key requirement for regulatory biowaivers under BCS Class II guidelines [134]. This suggests that surfactant–particle hybrids not only improve bioavailability but also promote formulation robustness under physiological variability.

Moreover, the integration of biopredictive dissolution media with PBPK (physiologically based pharmacokinetic) modeling is emerging as a powerful strategy to bridge preclinical performance with clinical outcomes. These tools help formulation scientists optimize hybrid systems not just for maximum solubility but for reliable systemic exposure, reinforcing the regulatory and translational credibility of these advanced delivery platforms. For instance, a PBPK modeling framework was employed to optimize a SEDDS–nanocrystal hybrid of fenofibrate, integrating in vitro dissolution data and absorption kinetics to accurately simulate human plasma profiles. This case exemplifies how PBPK models can support rational design decisions for surfactant–particle hybrid systems by bridging in vitro and in vivo observations [135].

### 5.2. Manufacturing and Regulatory Considerations

From a manufacturing standpoint, the development of surfactant–particle hybrid systems requires not only scientific optimization but also industrial scalability and process robustness. Techniques such as wet granulation, spray-drying, and hot-melt extrusion (HME) have proven effective for fabricating hybrids with acceptable flowability, compressibility, and solid-state stability [136]. However, critical formulation parameters—particularly drug loading, surfactant concentration, moisture content, and crystallinity—must be tightly controlled, as they directly influence both biopharmaceutical performance and manufacturability.

In modern formulation pipelines, these parameters are integrated into the Quality by Design (QbD) paradigm. Surfactants and particle engineering processes are now frequently defined as Critical Material Attributes (CMAs) and Critical Process Parameters (CPPs) within design-space models [137]. For instance, in HME-based solid dispersions, DoE modeling has shown that even small deviations in TPGS concentration or extruder temperature can lead to significant shifts in dissolution rate, crystallinity, or melt viscosity, which in turn affect process reproducibility and stability [138].

To ensure real-time control, Process Analytical Technology (PAT) tools—such as in-line NIR (Near-Infrared Spectroscopy), Raman spectroscopy, and focused beam reflectance measurement—are increasingly deployed. These allow continuous monitoring of key quality indicators like surfactant distribution, particle size, and amorphous content, which is especially vital in continuous manufacturing setups now being adopted for hybrid oral and injectable dosage forms [139].

From a regulatory perspective, surfactant–particle hybrids occupy a balancing position between established excipient use and emerging safety concerns. On one hand, the prior approval status of commonly used surfactants (e.g., Poloxamer 407, Cremophor RH40)—as listed in the FDA’s Inactive Ingredient Database (IID)—can facilitate regulatory submission by supporting safety arguments [140]. On the other hand, regulators are increasingly vigilant about surfactant-associated risks, including hypersensitivity, GI irritation, and bioaccumulation, especially in pediatric or chronic use populations [141].

Additional complexity arises when surfactants are paired with lipid-based carriers, as is common in solid SEDDS or nanostructured lipid carriers (NLCs). These systems may exhibit non-linear absorption, lymphatic transport, or supersaturation collapse, all of which can challenge bioequivalence demonstration. In such cases, clinical endpoint studies, fed/fasted comparisons, or innovator-matched dissolution profiles may be mandated by authorities [142].

Ultimately, successful translation of hybrid systems into commercial products hinges on balancing formulation innovation with regulatory pragmatism. The strategic use of QbD, PAT, and risk-based control systems enables formulators to achieve not only functional enhancement but also regulatory alignment. A cross-platform summary of these considerations—including critical quality metrics, common failure modes, and regulatory bottlenecks—is presented in Table 6, providing a practical reference for decision-making during formulation development and lifecycle management.

### 5.3. Safety and Excipient Acceptability

While many surfactants employed in hybrid systems carry GRAS (Generally Recognized As Safe) status, their safety profile is highly dose-dependent, especially when integrated into particle-engineered systems that amplify drug absorption efficiency. A prime example is Cremophor EL, which, despite its proven solubilization capacity, has been implicated in hypersensitivity, nephrotoxicity, and complement activation-related pseudoallergy (CARPA) reactions at elevated doses. These concerns have led to increased interest in alternatives such as Vitamin E TPGS, Poloxamer 188/407, or natural surfactants with better tolerability profiles [143].

Importantly, the permeability-enhancing functions of surfactants, while beneficial for certain poorly absorbed drugs, raise potential risks of non-selective barrier modulation. By disrupting tight junctions or altering membrane fluidity, surfactants may inadvertently permit the absorption of undesirable solutes such as bacterial toxins, allergens, or residual impurities. In animal studies, chronic oral exposure to bile salt derivatives like sodium taurocholate has led to mucosal erosion and epithelial thinning, although the human-safe thresholds for chronic use remain ill-defined [144].

To address these uncertainties, risk-based development strategies are essential. Regulatory bodies now recommend predictive pharmacokinetic modeling to estimate systemic surfactant exposure, combined with histopathological analysis in GLP-compliant toxicity studies—especially for novel excipient combinations or modified-release hybrid formulations [145]. These steps are particularly critical when surfactants are used outside established monograph limits, or in non-traditional routes such as pulmonary or intranasal administration.

Moreover, contextual safety considerations must guide excipient selection. Even well-established surfactants such as Polysorbate 80, while FDA-classified as GRAS, are subject to varying maximum allowable concentrations across regulatory jurisdictions. For instance, differences in allowable limits between the US FDA, EMA, and PMDA (Japan) complicate global development plans and necessitate region-specific formulation strategies. Pediatric and geriatric patients, who may exhibit altered mucosal integrity or impaired metabolic clearance, are particularly vulnerable to cumulative surfactant exposure. For example, reports of sorbitan monooleate (Span 80) accumulation in hepatic tissues following repeated administration in rodent models highlight the need for rigorous dose caps and long-term monitoring in sustained-release or chronic-use formulations [146].

One of the key advantages of hybrid strategies is their excipient-sparing potential. By leveraging particle size reduction or amorphization, it is often possible to achieve equivalent bioavailability with reduced surfactant load, thereby lowering toxicity risk and easing regulatory burden [8,147,148]. Nonetheless, excipient qualification must be tailored to the target patient population. In immunocompromised, pediatric, or polypharmacy settings, surfactant interaction with other drugs or altered barrier function must be considered to prevent unpredictable absorption or toxicity [46,149].

As safety concerns with synthetic surfactants persist, natural alternatives such as lecithin, saponins, or plant-derived glycosides are being increasingly investigated. These agents may offer improved biocompatibility and lower immunogenicity, but also present challenges such as batch-to-batch variability, limited scalability, and difficulty meeting GMP impurity standards [149,150].

A structured summary of these safety considerations—including toxicity findings, regulatory classification, and patient-specific risk profiles—is provided in Table 7, serving as a practical guide for rational excipient selection and dosage form design in surfactant–particle hybrid systems.

Regulatory acceptability of excipients also varies across regions. For instance, the FDA’s Inactive Ingredient Database (IID) restricts surfactant quantities based on precedent use in approved products, whereas the EMA requires explicit justification for excipients with known irritancy potential (e.g., PEG derivatives), often mandating warning labels. The PMDA in Japan follows stricter quantitative thresholds and may request excipient-specific toxicity data, even for GRAS-listed agents, particularly in pediatric indications. These regional discrepancies highlight the importance of early excipient planning in globally intended formulations.

## 6. Outlook

### 6.1. Strategic Advantages of Hybridization

The integration of surfactant systems with particle engineering techniques offers a compelling multidimensional strategy for overcoming long-standing challenges in oral and systemic drug delivery. Unlike traditional monolithic approaches—which focus either on solubilization or particle size modification—hybrid systems synergistically address both interfacial and structural limitations, enabling a layered enhancement of key biopharmaceutical attributes. This includes rapid dissolution, supersaturation maintenance, permeability modulation, and, increasingly, targeted biodistribution.

One of the most significant advantages of this dual-modality approach lies in its applicability to BCS Class II and IV compounds, where poor solubility and/or limited permeability restrict oral bioavailability. By simultaneously increasing the surface area through nanonization or dispersion and maintaining the drug in a solubilized state via micellar or interfacial stabilization, hybrid systems can achieve supersaturated yet kinetically stabilized microenvironments. These, in turn, generate favorable concentration gradients across epithelial barriers, leading to enhanced transcellular and paracellular absorption. Such strategies have not only improved C_max_ and AUC in vivo but have also been shown to reduce intra- and inter-subject variability, which is especially relevant in patient populations with variable GI transit times or compromised absorption windows.

Furthermore, hybridization can attenuate food effects, which often complicate oral dosing regimens and biowaiver approvals. The ability of surfactants to modulate bile salt interaction or facilitate lipid-mimetic absorption pathways, when combined with particle engineering that accelerates dissolution, leads to more predictable pharmacokinetics under both fasted and fed conditions. This predictability is of strategic importance in the context of global regulatory harmonization and bioequivalence testing.

In terms of platform versatility, hybrid systems offer an expansive formulation landscape—from surfactant-coated nanocrystals, to spray-dried SEDDS, to multi-layer solid dispersions—each customizable to specific route of administration, release profile, and patient population. The physical properties of these hybrids, including surface charge, mucoadhesion, hydrophobicity, and particle rigidity, can be precisely tuned for site-specific delivery or disease-state targeting. For example, modifying the surface of lipid-surfactant nanoparticles has enabled pulmonary and lymphatic delivery, while enteric-coated hybrid formulations have shown promise in colon-targeted therapeutics.

Emerging research also points to the use of these hybrid systems for biologics, including peptides, vaccines, and RNA therapeutics, where solubility, stability, and mucosal permeation remain key barriers. The protective matrix and solubilizing environment provided by hybrid platforms can shield labile molecules, prolong mucosal residence, and even enable non-invasive administration routes. With proper excipient optimization, these systems could play a pivotal role in extending oral delivery feasibility to traditionally injectable biologics [151].

Overall, surfactant–particle hybrid systems represent a forward-looking formulation paradigm. Their modular design enables mechanistic customization, route-specific adaptability, and patient-centric design, making them not just solubility enhancers, but integrative delivery platforms poised to address current and future therapeutic challenges across drug classes and clinical contexts.

### 6.2. Key Unresolved Challenges

Despite their substantial promise, surfactant–particle hybrid systems continue to face critical unresolved barriers that restrict their scalability, regulatory acceptance, and clinical reliability. One of the most pressing issues is long-term stability. Many hybrid systems—especially those incorporating amorphous drug forms or high surfactant content—remain vulnerable to recrystallization, phase separation, or chemical degradation, particularly under accelerated storage conditions involving humidity and heat. Although ICH guidelines provide general protocols for stability testing, there is no harmonized framework tailored to hybrid formulations, which often deviate from conventional solid or lipid dosage paradigms. This gap hinders shelf-life prediction and complicates global regulatory submissions, particularly for products intended for multiple climatic zones.

Another major challenge lies in the limited predictability of in vivo performance. Existing mechanistic models of supersaturation and micelle-mediated solubilization [152] often oversimplify the dynamic and heterogeneous gastrointestinal environment, failing to account for key physiological factors such as fluctuating bile salt levels, transient pH changes, and enzyme-mediated breakdown. As a result, in vitro–in vivo correlation (IVIVC) remains weak for many hybrid systems, necessitating time-consuming empirical testing. The absence of validated, biopredictive dissolution and permeation platforms impedes early-stage screening and prolongs formulation development timelines.

In addition, nonlinear surfactant–API interactions introduce formulation complexity. For example, surfactant concentrations above the critical micelle concentration (CMC) may paradoxically reduce permeability by trapping drugs in micelles, whereas concentrations below the CMC might fail to prevent precipitation altogether. These competing effects can lead to interpatient variability, unpredictable dose–exposure relationships, and safety concerns, especially for chronically administered or narrow therapeutic index drugs.

From a regulatory standpoint, hybrid systems often operate in gray zones of existing excipient and product classifications. Carriers such as lipid–surfactant nanosystems or multi-phase hybrid granules frequently fall outside current pharmacopeial monographs or lack clear regulatory precedent—particularly for non-oral routes like pulmonary or buccal delivery [153]. Furthermore, regional inconsistencies in surfactant limits, impurity thresholds, and solvent tolerances make global harmonization difficult, forcing developers to adopt region-specific formulation strategies that increase complexity and cost.

Finally, the fragmented nature of formulation development workflows remains a systemic bottleneck. Despite advances in QbD, DoE, PAT, and in silico modeling, these tools are often used in isolation rather than within an integrated, data-driven design framework. Without seamless integration of mechanistic databases, real-time analytics, and computational simulation, developers are left with inefficient, trial-based processes that reduce the scalability of hybrid platforms [154]. To fully realize their translational potential, hybrid systems require not only technological innovation but also infrastructure modernization, including standardized formulation decision trees, shared knowledge platforms, and cross-disciplinary alignment across formulation science, toxicology, and regulatory affairs.

### 6.3. Next-Generation Directions for Formulation Innovation

The future of hybrid drug delivery systems lies at the convergence of materials science, computational modeling, and personalized therapeutics, driving a shift from empirical formulation practices toward integrated, model-informed development paradigms. Emerging technologies—particularly artificial intelligence (AI) and machine learning (ML)—hold the potential to revolutionize formulation design by leveraging large datasets that link formulation attributes with biopharmaceutical performance. These algorithms can be embedded directly into Quality by Design (QbD) workflows to predict optimal excipient compositions, particle size distributions, and release profiles with increasing precision [155]. Recent reports show that AI-assisted formulation selection can reduce development timelines by up to 40%, particularly in early-stage screening of surfactants and stabilizers [156].

A typical implementation begins with comprehensive data acquisition, encompassing raw material characterization, high-throughput solubility and permeability screening, and in vitro–in vivo correlation (IVIVC) studies. These datasets can then inform AI/ML models that account for formulation variables and processing conditions defined through Design of Experiments (DoE). Beyond prediction, digital twin simulations of manufacturing lines enable real-time forecasting of Critical Quality Attributes (CQAs) and potential failure modes such as crystallization or phase separation. This model-based approach allows for iterative refinement and preemptive risk mitigation before scale-up [157]. Incorporating AI-guided digital twins into continuous manufacturing has also shown benefits in scaling hybrid systems with complex surfactant–drug interactions [158].

Once an optimal formulation is identified, pilot-scale continuous manufacturing—augmented with real-time Process Analytical Technology (PAT) tools such as in-line NIR spectroscopy, Raman mapping, or focused beam reflectance—can support adaptive control of Critical Process Parameters (CPPs). Such closed-loop systems reduce batch variability and support rapid transition from laboratory concept to GMP-compliant production. This workflow is particularly advantageous for narrow therapeutic index (NTI) drugs, where small deviations in dose or release profile can have significant clinical implications. For NTI drugs formulated as surfactant–particle hybrids, even small pH variations during coating require active monitoring via PAT to ensure content uniformity [159].

Hybrid strategies also align closely with the paradigm of personalized medicine. Particle morphology, surfactant functionality, and release kinetics can be tailored based on individual patient parameters, such as genetic polymorphisms, drug metabolism profiles, or gut microbiome composition. For example, pharmacogenomic screening could inform surfactant selection to avoid hypersensitivity risks, while microbiota-informed design could guide the use of pH-sensitive or enzymatically activated coatings for site-specific drug release [160]. Physiologically based pharmacokinetic (PBPK) modeling further enables the simulation of drug exposure across population subgroups, supporting rational dose adjustments and formulation optimization early in development.

Despite these advances, current PBPK and IVIVC models face limitations when applied to multicomponent hybrid systems. The complexity arising from surfactant–excipient interactions, dynamic precipitation, and gut-environment-dependent permeability modulation often exceeds the scope of standard models. As such, more modular and adaptive modeling tools are needed to account for these nonlinear and emergent behaviors in hybrid formulations [161].

Looking ahead, innovation is expected in stimuli-responsive hybrid platforms that enable spatiotemporal control of drug release. This includes pH-triggered surfactant assemblies, thermoresponsive nanocrystals, and enzyme-activated lipid–surfactant carriers, which can adjust drug delivery in response to local physiological cues [162]. When integrated with wearable biosensors or implantable monitoring devices, these systems could form the basis of closed-loop therapeutic platforms, capable of autonomously adjusting drug release based on real-time physiological signals such as inflammation, glucose levels, or pH. While these advanced systems require rigorous validation and regulatory alignment, they represent the next frontier of hybrid formulation—intelligent, adaptable, and precision-tailored to individual patient needs.

To fully realize these innovations, regulatory frameworks must evolve to include model-informed approval pathways. The integration of QbD, AI, and PAT is being actively explored by regulatory bodies such as the FDA and EMA to support real-time release testing (RTRT) and continuous verification. Future hybrid formulations may thus benefit from more adaptive, data-driven lifecycle management.

## 7. Conclusions

The convergence of surfactant science and particle engineering has catalyzed a fundamental shift in oral drug delivery, offering a robust framework for overcoming the solubility and permeability limitations that have historically constrained the bioavailability of many therapeutics. By uniting complementary mechanisms—ranging from interfacial energy modulation and micellar solubilization to particle size reduction and solid-state manipulation—hybrid systems provide a multidimensional platform capable of enhancing dissolution, sustaining supersaturation, and improving membrane transport.

This review has critically examined the classification, mechanistic functions, and formulation strategies of surfactants, alongside core particle engineering technologies such as nanocrystallization, solid dispersion, and spray-based drying. It further demonstrated how integration of these approaches can produce tailored, high-performance systems suitable for a wide range of biopharmaceutical challenges. Importantly, we explored not only the functional benefits but also the practical constraints, manufacturing considerations, and safety implications of these hybrid technologies—elements that are often underemphasized in previous reviews.

Despite their significant promise, surfactant–particle hybrid systems still face unresolved hurdles. Long-term physical stability, especially under accelerated or real-world storage conditions, remains a central concern. Additionally, the lack of predictive in vitro–in vivo models for hybrid behavior—particularly under dynamic gastrointestinal conditions—continues to limit rational formulation design. Regulatory alignment is also fragmented, with differing global expectations for excipient limits, residual solvents, and biowaiver criteria complicating cross-market development.

Looking forward, the evolution of these systems is likely to be shaped not only by advances in material science but also by digital formulation tools, continuous manufacturing technologies, and patient-specific design strategies. The intersection of hybrid platforms with AI-driven modeling, real-time process analytics, and personalized medicine represents a transformative frontier—one that holds the potential to move beyond empirical formulation toward data-informed, adaptable therapeutics.

Ultimately, surfactant–particle hybrid systems represent more than an incremental improvement in solubility enhancement—they embody a strategic integration of physicochemical innovation and translational feasibility. Their future success, however, will depend on the development of systematic approaches to address their key limitations. Only through such multidisciplinary integration can these promising technologies mature from niche applications into widely adopted solutions that meaningfully expand the therapeutic landscape for poorly soluble and biopharmaceutically challenging drugs.

## Figures and Tables

**Table 1:** Classification and characteristics of common pharmaceutical surfactants.

Surfactant	HLB Value	Physicochemical Properties	Regulatory Classification
Polysorbate 80 (Tween 80)	15.0	Non-ionic, high solubility in water, emulsifier	GRAS, USP/NF listed, widely accepted in oral/parenteral
Sodium Lauryl Sulfate (SLS)	40.0	Anionic, strong foaming agent, detergent properties	FDA-approved within limits, used in oral/dermal
Poloxamer 188	29.0	Non-ionic block copolymer, thermoresponsive	GRAS, used in oral and injectable formulations
Poloxamer 407	22.0	Thermoreversible gelation, non-ionic surfactant	GRAS, used in gels and ophthalmics
Cremophor EL	14.0	Non-ionic, derived from castor oil, solubilizer	FDA inactive ingredients list, parenteral use caution
PEG 400	11.5	Low-molecular PEG, solvent and solubilizer	GRAS, oral and parenteral solvent
Span 20	8.6	Lipophilic surfactant, emulsifier	FDA listed, cosmetic and pharma
Span 60	4.7	Waxy emulsifier, low solubility	Generally recognized as safe
Span 80	4.3	Lipophilic, emulsifier for oils	GRAS, widely used emulsifier
Lecithin	7.0	Phospholipid, natural emulsifier	Natural origin, safe for oral use
Brij 35	16.9	Polyoxyethylene ether, solubilizer	Pharmaceutical excipient, widely accepted
Brij 58	15.7	Polyoxyethylene ether, mild surfactant	Safe non-ionic for topical and oral use
Pluronic F68	29.0	Thermoresponsive, low toxicity	Used in IV and oral formulations
Pluronic F127	22.0	Micelle forming, thermoresponsive	Pharmaceutical grade, injectable
Polysorbate 20 (Tween 20)	16.7	Non-ionic, emulsifier, used in food and pharma	GRAS, oral and topical
Polysorbate 60 (Tween 60)	14.9	Emulsifier, stabilizer	GRAS, used in foods and drugs
Sodium deoxycholate	18.0	Bile salt, solubilizing agent	FDA approved bile salt
Sodium taurocholate	38.0	Bile salt, stabilizer	Oral formulation enhancer, FDA listed
Sodium cholate	18.0	Bile salt, enhances solubilization	Oral solubilizer, pharmacopoeial grade
Sodium glycocholate	23.1	Bile salt, amphipathic	Generally safe, bile origin
Lauryl glucoside	13.4	Non-ionic, biodegradable	Used in natural cosmetics
Caprylocaproyl polyoxylglycerides	12.0	Medium-chain PEG glyceride	Pharmacopoeial excipient, GRAS
PEG-6 caprylic/capric glycerides	12.0	Low toxicity, used in lipid-based formulations	GRAS, oral formulations
Myristyl lactate	7.5	Ester of lactic acid, skin conditioning	Cosmetic and topical approved
PEG-40 hydrogenated castor oil	14.0	Castor oil derivative, emulsifier	GRAS, oral and parenteral
PEG-100 stearate	18.8	Stearic acid ester, solubilizer	GRAS, safe emulsifier
PEG-32 stearate	15.2	Stearic acid PEG ester, co-emulsifier	Pharma and cosmetic use
PEG-20 methyl glucose sesquistearate	14.9	Non-ionic emulsifier, skin compatible	Low toxicity, GRAS listed
PEG-60 almond glycerides	11.5	Emollient, mild solubilizer	Mild, cosmetic and pharma
PEG-75 lanolin	10.5	Lanolin derivative, solubilizer	Widely accepted cosmetic emulsifier
Cocamidopropyl betaine	10.0	Zwitterionic, gentle on skin	GRAS, topical use
Cocamide DEA (Diethanolamine)	11.0	Coconut oil-derived, foam booster	FDA approved topical use
Oleth-10	12.5	Ether-based, mild surfactant	Used in pharma and cosmetics
Oleth-20	15.0	Polyethylene glycol ether, solubilizer	Listed as inactive ingredient
Decyl glucoside	13.6	Sugar-based, mild non-ionic surfactant	Approved for dermal use
Lauryl glucoside	13.4	Glucoside-based, non-ionic, low irritation	GRAS, used in baby products
Caprylyl glycol	11.5	Multifunctional solubilizer	Safe in pharma and dermal
Glyceryl oleate	4.3	Non-ionic emulsifier	Cosmetic emulsifier
Sorbitan trioleate	1.8	Oil-soluble emulsifier	GRAS emulsifier
Sorbitan monopalmitate	6.7	Waxy, low HLB emulsifier	Food additive and emulsifier
Ethoxydiglycol	9.8	Hydrophilic solvent	Cosmetic excipient
Sucrose laurate	21.0	Sucrose ester, non-ionic	GRAS surfactant
Sodium lauroyl lactylate	7.2	Mild surfactant, food grade	Used in food and pharma
PEG-12 dimethicone	11.0	Silicone-based surfactant	GRAS listed
PEG-7 glyceryl cocoate	14.0	Coconut-derived solubilizer	Cosmetic solvent/emulsifier
Capmul MCM	14.0	Medium chain mono/diglycerides	Topical and oral accepted
Labrasol	9.0	PEG caprylic/capric glyceride	Oral lipid carrier
Labrafil M 2125 CS	4.0	Lipophilic PEG derivative	Oral bioavailability enhancer
Transcutol P	4.5	Solvent, enhancer	GRAS listed

**Table 2:** Comparative overview of particle engineering techniques.

Technique	Particle Size Range	Thermodynamic Stability	Scalability	Major Limitations	Target API Type	References
Micronization (Jet Milling)	1–10 µm	Low	High	Poor flow, electrostatic charge	Hydrophobic APIs	[60,61,62]
Nanocrystallization (Top-down)	<1 µm	Moderate	Moderate	Ostwald ripening, sedimentation	Poorly soluble APIs	[64,65,66]
Nanocrystallization (Bottom-up)	<1 µm	Moderate	Moderate	Reproducibility, scale-up	BCS Class II APIs	[64,65]
Spray-Drying	1–5 µm	Moderate	High	Thermal stress, solvent residue	Thermostable APIs	[68]
Freeze-Drying (Lyophilization)	1–10 µm	High	Low	High cost, long duration	Thermolabile APIs	[68,69]
Amorphous Solid Dispersion (Hot melt)	Amorphous	Moderate	Moderate	Thermal degradation	BCS II/IV	[70]
Amorphous Solid Dispersion (Spray dried)	Amorphous	Moderate	High	Recrystallization risk	BCS II drugs	[70,71]
Antisolvent Precipitation	50–500 nm	Low	Moderate	Solvent removal	Hydrophobic APIs	[64]
Supercritical Fluid Processing	0.1–5 µm	High	Low	Expensive equipment	Heat-sensitive APIs	[60]
Fluidized Bed Coating	1–100 µm	High	High	Layer uniformity	Taste masking, controlled release	[63]
Spray Chilling/Cooling	10–100 µm	High	High	Particle aggregation	Lipid-based APIs	[60]
Cryomilling	1–5 µm	Moderate	Low	Cold handling required	Heat-sensitive APIs	[67]

**Table 3:** Refined summary of surfactant–particle hybrid techniques.

Hybrid Platform	Key API Examples	Surfactant Types	Engineering Technique	Performance Advantage	References
Surfactant-assisted wet granulation	Telmisartan, Ritonavir, Naproxen	Polysorbate 80, PEG 400, Sodium lauryl sulfate (SLS)	High-shear wet granulation using surfactant-containing binder solution	Improves powder wettability and granule compressibility; enhances drug release and disintegration; enables low surfactant dose	[95,96,97,98]
Surfactant-coated nanocrystals	Curcumin, Silymarin, PLGA-loaded Dexamethasone	Poloxamer 407, Sodium deoxycholate, TPGS	Wet milling or high-pressure homogenization followed by surfactant adsorption	Enhances redispersibility, colloidal stability, and membrane interaction; suitable for mucosal delivery and pediatric forms	[99,100]
Solid dispersions with surfactants	Itraconazole, Ritonavir, Tadalafil	Cremophor RH40, Solutol HS15, Vitamin E TPGS	Hot-melt extrusion or spray-drying to form amorphous ternary systems	Maintains supersaturation; reduces recrystallization; improves physical stability and bioavailability in fed/fasted states	[103,104,105]
Solidified SEDDS	Ritonavir, Cyclosporine, Tacrolimus	Poloxamer 188, Tween 80, Labrasol	Spray-drying of SEDDS onto porous carriers like Neusilin or Aerosil	Improves storage stability and redispersibility; enhances lymphatic transport; suitable for high log P drugs	[106,107,108,109,110]
Lipid–surfactant hybrid systems (NLCs)	Quercetin, Resveratrol, Paclitaxel	Polysorbate 80, TPGS, Cremophor EL	Lipid phase preparation followed by ultrasonication and surfactant coating	Enhances permeability and tissue targeting via surfactant-mediated surface modulation; allows dual drug loading	[111,112,113,114]
Spray freeze-drying with surfactants	Voriconazole, Salmon Calcitonin, Influenza vaccines	SLS, Lecithin, Tween 20	Atomization into liquid nitrogen and lyophilization for porous hybrid microparticles	Enables pulmonary or mucosal delivery of fragile biomolecules; offers rapid dissolution and thermal protection	[115,116,117,118]

**Table 4:** Summary of manufacturability, stability, and safety of surfactant–particle hybrid systems.

Hybrid System	API Load Suitability	Manufacturing Feasibility	Stability Challenges	Safety Considerations
Micronization	Moderate	High (conventional equipment)	Moisture sensitivity	Low
Nanocrystals	High	Medium (coating required)	Aggregation, Ostwald ripening	Moderate (surfactant exposure)
Solid Dispersions	Low–Moderate	High (HME/spray-drying)	Recrystallization, surfactant migration	Low–Moderate
SEDDS Hybrids	High	Medium (adsorption/spray-drying)	Reconstitution failure, phase separation	Moderate (surface migration)
NLC Hybrids	Moderate	Medium (emulsification)	Surfactant desorption, polymorphism	Moderate–High (immunogenicity)
Spray Freeze-Drying	Low	Low (cryogenic process)	Moisture uptake, oxidative degradation	Low–Moderate (route dependent)

**Table 5:** Summary of drug release mechanisms in surfactant–particle hybrid systems.

Hybrid Type	Drug Release Mechanism	Mechanistic Highlights
Surfactant-coated Nanocrystals	Surface erosion and diffusion	Surfactants stabilize nanocrystals and enhance wetting; enables rapid dissolution.
Amorphous Solid Dispersions with Surfactants	Supersaturation followed by precipitation inhibition	Surfactants maintain supersaturation and prevent recrystallization.
SEDDS Hybrids	Emulsification followed by lipid digestion	Surfactants aid in droplet formation; supports lymphatic uptake.
NLC Hybrids	Diffusion through lipid matrix	Lipid matrix controls release; surfactants enhance permeability and mucosal uptake.
Spray Freeze-Dried Hybrids	Burst release followed by diffusion	Highly porous structure enables fast disintegration and immediate drug release.

**Table 6:** Manufacturing and regulatory considerations for surfactant–particle hybrids.

Aspect	Key Considerations	Examples/Tools	References
Scalability	Process must tolerate industrial-scale loads, maintain particle size and homogeneity.	Wet granulation, spray-drying, hot-melt extrusion (HME); scalability trials.	[137]
Critical Quality Attributes (CQAs)	Drug content uniformity, particle size distribution, surfactant level, polymorphic form.	Design of Experiments (DoE), QbD risk assessment matrices.	[138,139]
Process Monitoring	Real-time monitoring of particle morphology, surfactant distribution, crystallinity.	PAT tools: in-line NIR, Raman spectroscopy, focused beam reflectance measurement.	[140]
Regulatory Approval	Use of excipients with prior precedence; evaluation of surfactant toxicology and stability.	FDA IID surfactant listing, GRAS status, excipient compatibility studies.	[141,142]
Toxicological Limits	Surfactant use limits in chronic or pediatric settings; GI and hypersensitivity issues.	Risk assessments, NOAEL (No-Observed-Adverse-Effect Level) studies, pediatric extrapolation guidelines.	[142]
Bioequivalence Challenges	Surfactant-lipid hybrids may alter absorption; nonlinear kinetics; lymphatic uptake.	Clinical endpoint studies, biowaivers, dissolution profile matching.	[143]
Manufacturing Robustness	Batch-to-batch reproducibility; control of CPPs such as drying temperature, extrusion speed.	HME barrel temperature monitoring, fluid bed granulation sensors.	[137,139]
Formulation Stability	Hygroscopicity, surfactant migration, recrystallization on storage.	Stability chambers, PXRD (Powder X-Ray Diffraction)/DSC (Differential Scanning Calorimetry) analysis, ICH (International Council for Harmonisation) Q1A(R2) compliance.	[140,141]

**Table 7:** Safety considerations and regulatory risks of common surfactants in hybrid drug delivery systems.

Surfactant (Type)	Key Safety Concerns	Mechanism of Concern	Populations at Risk	Regulatory Remarks	References
Cremophor EL (non-ionic)	Hypersensitivity, nephrotoxicity	Histamine release, renal accumulation	General population, especially IV use	Phased out in some injectable products, replaced by safer excipients	[144]
Vitamin E TPGS (amphiphilic)	Generally safe, but accumulation risk at high dose	Biliary clearance saturation	Pediatric, hepatic impairment	Used widely in oral formulations, QbD-preferred	[144,148]
Poloxamers (188, 407) (non-ionic)	GI discomfort, systemic absorption in neonates	Altered membrane fluidity	Neonates, elderly	Listed in FDA IID; max allowable concentrations vary	[144,147]
Sodium taurocholate (bile salt)	Epithelial erosion, tight junction damage	Permeation enhancer disrupting barrier integrity	Chronic use, GI-compromised	Thresholds undefined in humans, known toxic in animals	[145]
Span 80 (Sorbitan monooleate)	Hepatic accumulation in long-term exposure	Lipid accumulation	Chronic oral administration	Low-dose recommended in sustained-release platforms	[147]
Polysorbate 80 (non-ionic)	Oxidative degradation, hypersensitivity	Auto-oxidation, histamine release	Parenteral, elderly	GRAS by FDA, but global variability in limits (EU, JP, CN)	[144,150]
Lecithin (natural phospholipid)	Variable purity, allergenicity	Source-dependent variability	Immunocompromised, allergic patients	Natural, but batch variability limits scalability	[151]
Saponins (natural)	Hemolysis, immune stimulation	Membrane disruption, adjuvant effect	Not safe for IV, caution in oral	Still under exploration, no global monograph yet	[152]

## Data Availability

No new data were created or analyzed in this study. Data sharing is not applicable to this article.

## Abbreviations

The following abbreviations are used in this manuscript:

GRAS	Generally Recognized As Safe
HLB	Hydrophilic-Lipophilic Balance
USP	United States Pharmacopeia
NF	National Formulary
FDA	Food and Drug Administration
PEG	Polyethylene Glycol
DEA	Diethanolamine
IV	Intravenous
API	Active Pharmaceutical Ingredient
BCS	Biopharmaceutics Classification System
PLGA	Poly(lactic-co-glycolic acid)
TPGS	D-α-Tocopheryl Polyethylene Glycol 1000 Succinate
SEDDS	Self-Emulsifying Drug Delivery System
NLCs	Nanostructured Lipid Carriers
QbD	Quality by Design
PAT	Process Analytical Technology
NIR	Near-Infrared Spectroscopy
FDA IID	FDA Inactive Ingredient Database
NOAEL	No-Observed-Adverse-Effect Level
CPPs	Critical Process Parameters
PXRD	Powder X-Ray Diffraction
DSC	Differential Scanning Calorimetry
ICH	International Council for Harmonisation
GI	Gastrointestinal
